# Effect of 6-Month HIV Preexposure Prophylaxis Dispensing With Interim Self-testing on Preexposure Prophylaxis Continuation at 12 Months: A Randomized Noninferiority Trial

**DOI:** 10.1001/jamanetworkopen.2023.18590

**Published:** 2023-06-15

**Authors:** Katrina F. Ortblad, Ashley R. Bardon, Peter Mogere, Catherine Kiptinness, Stephen Gakuo, Sarah Mbaire, Katherine K. Thomas, Nelly R. Mugo, Jared M. Baeten, Kenneth Ngure

**Affiliations:** 1 Public Health Sciences Division, Fred Hutchinson Cancer Center, Seattle, Washington; 2 Department of Global Health, University of Washington, Seattle; 3 Department of Epidemiology, University of Washington, Seattle; 4 Center for Clinical Research, Kenya Medical Research Institute, Nairobi, Kenya; 5 Department of Medicine, University of Washington, Seattle; 6 School of Public Health, Jomo Kenyatta University of Agriculture and Technology, Nairobi, Kenya; 7 now with Gilead Sciences, Foster City, California

## Abstract

**Importance:**

Daily oral HIV preexposure prophylaxis (PrEP) delivery requires quarterly clinic visits for HIV testing and drug refilling that are costly to health systems and clients.

**Objective:**

To evaluate whether 6-month PrEP dispensing supported with interim HIV self-testing (HIVST) results in noninferior PrEP continuation outcomes at 12 months compared with standard quarterly clinic visits.

**Design, Setting, and Participants:**

This randomized noninferiority trial was conducted from May 2018 to May 2021 with 12 months of follow-up among PrEP clients aged 18 years or older who were returning for their first refill at a research clinic in Kiambu County, Kenya.

**Intervention:**

Participants were randomized 2:1 to (1) 6-month PrEP dispensing with semiannual clinic visits and interim HIVST at 3 months or (2) standard-of-care (SOC) PrEP delivery with 3-month dispensing, quarterly clinic visits, and clinic-based HIV testing.

**Main Outcomes and Measures:**

Prespecified 12-month outcomes included recent HIV testing (any in past 6 months), PrEP refilling, and PrEP adherence (detectable tenofovir-diphosphate concentrations in dried blood spots). Binomial regression models were used to estimate risk differences (RDs), and a 1-sided 95% CI lower bound (LB) of −10% or greater was interpreted as noninferior.

**Results:**

A total of 495 participants were enrolled, with 329 enrolled in the intervention group and 166 enrolled in the SOC group; 330 (66.7%) were women, 295 (59.6%) were in serodifferent relationships, and the median (IQR) age was 33 (27-40) years. At 12 months, 241 individuals in the intervention group (73.3%) and 120 in the SOC group (72.3%) returned to clinic. In the intervention group, recent HIV testing was noninferior (230 individuals [69.9%]) compared with the SOC group (116 [69.9%]; RD, −0.33%, 95% CI LB, −7.44%). PrEP refilling in the intervention group (196 [59.6%]) was inconclusive compared with the SOC group (104 [62.7%]; RD, −3.25%; 95% CI LB, −10.84%), and PrEP adherence was noninferior in the intervention group (151 [45.9%]) compared with the SOC group (70 [42.2%]; RD, 4.96%; 95% CI LB, −2.46%). No HIV seroconversions were observed over the follow-up period.

**Conclusions and Relevance:**

In this analysis of secondary trial end points at 1 year, semiannual PrEP dispensing with interim HIVST resulted in noninferior recent HIV testing and PrEP adherence compared with SOC quarterly PrEP dispensing. This novel model has the potential to optimize PrEP delivery.

**Trial Registration:**

ClinicalTrials.gov Identifier: NCT03593629

## Key Points

**Question:**

Does 6-month daily oral HIV preexposure prophylaxis (PrEP) dispensing supported with interim HIV self-testing reduce the number of annual PrEP clinic visits without jeopardizing client continuation outcomes at 12 months?

**Findings:**

In a randomized noninferiority trial among 495 adults in Kenya, semiannual PrEP dispensing supported with interim HIV self-testing resulted in noninferior HIV testing and PrEP adherence 1 year after initiation compared with standard quarterly PrEP dispensing.

**Meaning:**

The findings of this study suggest that this differentiated model of PrEP service delivery could simplify and optimize PrEP delivery.

## Teaser

This randomized noninferiority trial evaluated whether 6-month preexposure prophylaxis (PrEP) dispensing, supported by interim HIV self-testing, results in noninferior PrEP continuation outcomes at 12 months vs standard quarterly clinic visits.

## Introduction

Differentiated models of service delivery that can decongest overcrowded clinics, can bolster self-care, and are patient-centered are needed to increase delivery efficiencies and engagement with HIV preexposure prophylaxis (PrEP) services.^1^ Since the World Health Organization recommended tenofovir-based daily oral PrEP as an HIV prevention option in 2015,^2^ the number of individuals initiating PrEP has been steadily increasing,^3^ but many populations that could benefit from PrEP services remain unserved^3^ or struggle with continued engagement in PrEP care.^4^ In many settings, the dominant model of oral PrEP delivery is at HIV clinics within health care facilities, where quarterly PrEP clinic visits are required for HIV testing (to detect potential breakthrough infections) and associated counseling services.^5^ However, barriers to PrEP delivery and access in this setting exist, including system-level (eg, costs), clinician-level (eg, time serving clients, competing priorities), and client-level (eg, time waiting at and traveling to facilities, stigma associated with visits) barriers.^6,7,8^

HIV self-testing (HIVST) is a relatively new technology that has the potential to simplify and address barriers to clinic-delivered PrEP services. For example, HIVST could be used by clients while waiting to reduce their time spent at clinics^9,10^ or to replace clinic visits that are required for quarterly HIV testing.^11,12^ A recent systematic literature review on HIVST-supported models of PrEP delivery^13^ found that these models were largely perceived as feasible and acceptable among PrEP clinicians and clients, but robust evidence on their effectiveness was limited, and the limited effectiveness studies only measured short-term PrEP continuation outcomes (ie, engagement in care ≤6 months). Thus, the potential for HIVST-supported models of PrEP delivery to increase delivery efficiencies while sustaining clients’ long-term engagement in PrEP services beyond 6 months—one of the greatest challenges with PrEP service delivery—remains unknown.

In this study, we tested the effect of an HIVST-supported PrEP delivery model on PrEP continuation outcomes in Kenya. Specifically, we tested a model that used HIVST to reduce the number of annual clinic visits for PrEP in half, by enabling 6-month PrEP dispensing supported with at-home HIVST between semiannual clinic visits. We hypothesized that despite the fewer touch points with the health system and associated counseling opportunities, this model of PrEP delivery would result in noninferior PrEP continuation outcomes (eg, recent HIV testing, PrEP refilling, and PrEP adherence) for clients interested in continued PrEP services 1 year later compared with the standard model of PrEP delivery with quarterly clinic visits.

## Methods

### Study Design

JiPime-JiPrEP, which means “test yourself, PrEP yourself” in Kiswahili, the language spoken by Swahili populations in Kenya, was an individually randomized, unblinded, noninferiority implementation trial testing the effect of 6-month PrEP dispensing supported with HIVST on PrEP continuation outcomes.^11^ The effects of this intervention on recent HIV testing, PrEP refilling, and PrEP adherence at 6 months have been reported elsewhere^12^; the present analysis reports effects 1 year following enrollment. The trial was conducted at the Partners in Health and Research Development research clinic in Thika, Kenya, a peri-urban town in Kiambu County (population-level HIV prevalence of approximately 4%), approximately 40 km from the capital city Nairobi.^13^ This report follows the Consolidated Standards of Reporting Trials (CONSORT) reporting guidelines for parallel-group randomized trials.^14^ The trial protocol^11^ was approved by the Scientific Ethics Review Unit at the Kenya Medical Research Institute and the Human Subjects Division at the University of Washington (Supplement 1). All eligible participants were enrolled by research staff and provided written informed consent. We had a data safety monitoring board (DSMB) for this trial, which met 3 times over the trial duration.

### Participants

**Figure 1.**
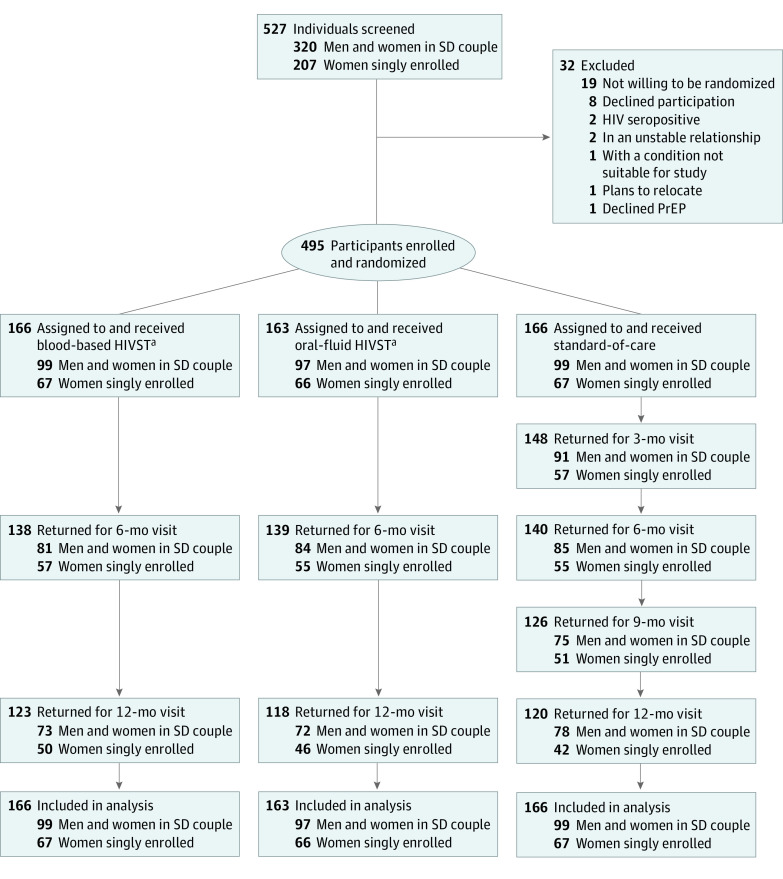
Study Flow Diagram SD indicates serodifferent. ^a^Participants randomized to these intervention groups received 6-month preexposure prophylaxis (PrEP) dispensing with HIV self-testing (HIVST) (either oral-fluid or blood-based) and semiannual clinic visits.

From May 28, 2018, to February 24, 2020, we recruited 495 PrEP clients who were returning for a scheduled follow-up visit 1 month after initiating PrEP from existing HIV counseling and testing centers and HIV clinics (Figure 1). We recruited 2 different populations that could benefit from PrEP services: (1) 295 individuals in HIV serodifferent couples (165 men and 130 women) and (2) 200 women not in known HIV serodifferent couples (ie, singly enrolled). Individuals were eligible for participation if they were aged 18 years or older, HIV-negative (confirmed via rapid testing according to national guidelines),^5^ refilling PrEP 1 month after initiation with the intention to continue use, not participating in another HIV prevention trial, and willing to be randomized to the study intervention.

### Randomization

At enrollment, participants were randomized 2:1 to an intervention group (of which there were 2, with an additional 1:1 assignment) or the control group using opaque envelopes opened in the presence of research staff. The randomization list was prepared using variable block sizes by the study statistician (K.K.T.), who was not otherwise involved in participant contact. Participants and study staff were unmasked to the randomization assignment due to the nature of the intervention.

### Procedures

We randomized participants to the following groups: (1) 6-month PrEP dispensing with HIVST, which included a 6-month drug supply, 2 HIVST kits, and semiannual clinic visits or (2) standard of care (SOC) PrEP dispensing, which included a 3-month drug supply, no HIV self-tests, and quarterly clinic visits. Participants in the intervention group were further randomized to receive either 2 blood-based or 2 oral-fluid HIVST kits. All participants received a PrEP supply of tenofovir disoproxil fumarate/emtricitabine or lamivudine (TDF/FTC or 3TC). Participants in the SOC group were scheduled for quarterly clinic visits (at 3, 6, 9, and 12 months), and those in the intervention group were scheduled for semiannual clinic visits (at 6 and 12 months).

Participants in the intervention group randomized to blood-based HIVST received Mylan Rapid HIV 1&2 tests (Mylan Lab Ltd), and those randomized to oral-fluid HIVST received OraQuick Rapid HIV-1/2 Antibody Tests (OraSure Technologies). At enrollment, all participants in the intervention group received training by study staff on the HIVST process, HIVST informational brochures (in English and Kiswahili), and a toll-free helpline phone number for 24-hour, on-demand support (if needed); they were encouraged to use 1 HIV self-test midway between PrEP clinic visits (ie, 3 months following refilling) and the other as desired (ie, for back-up testing, partner testing, or testing between clinic visits).

At every scheduled clinic visit, we delivered PrEP services following the 2018 Kenya National PrEP Guidelines^5^: counseling on HIV risk reduction strategies and the importance of PrEP adherence, assessment of potential PrEP adverse effects, syndromic assessment of sexually transmitted infections, urine pregnancy testing (women), rapid diagnostic HIV testing, and PrEP dispensing (per randomization group). Additionally, serum creatinine clearance testing was completed at enrollment and semiannually. For participants in HIV serodifferent couples, those who had been using PrEP for 6 months and reported their partner had been receiving antiretroviral therapy consistently for 6 months were recommended to discontinue PrEP, per national guidelines.^5^ We followed standard clinical procedures for antiretroviral treatment appointment reminders in Kenya: a phone call 1 day following a missed scheduled clinic visit and a second phone call 7 days following the first call.

Trained research assistants completed electronic questionnaires (CommCare [Dimagi]) with participants at each clinic visit. The questionnaires included information on participants’ demographic characteristics, use of HIV testing (including HIVST), health-seeking and sexual behaviors, and HIV testing preferences. At the 6- and 12-month clinic visits, we also collected dried blood spot (DBS) samples from each participant for biologic adherence measurements.

### Outcomes

We prespecified measuring outcomes of successful PrEP continuation (ie, HIV testing, PrEP refilling, and PrEP adherence) 12 months following randomization. For HIV testing, we measured (1) recent HIV testing, defined as any self-reported testing in past 6 months, and (2) continuous HIV testing, defined as testing 3 or more times since randomization (once between randomization and the 6-month clinic visit [self-reported], once at the 6-month visit [clinic records], and once between the 6- and 12-month visits [self-reported]). For PrEP refilling, we measured (1) refilling at the 12-month visit and (2) refilling at both the 6- and 12-month visits (both measured using pharmacy dispensing records). For PrEP adherence, we measured (1) adherence at the 12-month visit and (2) adherence at both 6- and 12-month visits. We categorized any detectable tenofovir-diphosphate (TFV-DP) concentration in DBS, measured using liquid chromatography tandem mass spectrometry,^15^ as indicative of PrEP adherence.

All study outcomes were binary and dependent on participants’ retention in care at the 6- and 12-month clinic visits. We used inclusive retention windows for each visit that went until 2 weeks before the start of participants’ next scheduled visit, resulting in different retention windows for participants in the intervention and SOC groups at 6 months. For the 12-month visit, participants were eligible for follow-up for up to 15 months.

### Statistical Analysis

All our analyses were intention-to-treat, and we assumed that participants lost to follow-up did not achieve the outcomes of interest. We prespecified our primary comparison to be the intervention group (including both forms of HIVST) vs the SOC group because we hypothesized that the effect of the intervention would be related to the frequency of PrEP clinic visits not the HIVST modality. However, we also compared the blood-based HIVST and the oral-fluid HIVST intervention groups individually with the SOC group to understand whether there were differences in testing modality.

We measured the proportion of participants with each outcome by group and estimated risk differences (RDs) and 1-sided 95% CI lower bounds (LBs) using binomial regression models with identity link, adjusted for study population (eg, men in serodifferent couples, women in serodifferent couples, and women singly enrolled). In keeping with a noninferiority trial design, we prespecified a noninferiority margin of −10% and considered the intervention group noninferior to the SOC group for outcomes with a 1-sided 95% CI LB of −10% or greater.^16^ The study sample size calculations were based on the PrEP adherence primary outcome measured at 6 months; 495 participants provided 74% power to rule out a 10% difference between the intervention and SOC groups, assuming 80% adherence, 10% loss to follow-up, and that missing results did not achieve the outcomes (a suggestion by our DSMB following trial initiation, which resulted in adjustments to our original calculations).^12^ All analyses were conducted in StataBE version 17 (StataCorp).

We conducted prespecified subgroup analyses and additional sensitivity analyses. Subgroup analyses were conducted to identify any marked outcome differences for (1) individuals in HIV serodifferent couples (noninferiority analysis) and (2) women singly enrolled (superiority analysis), populations we hypothesized might have different PrEP continuation needs at 12 months. For the subgroup of women singly enrolled, we prespecified a superiority analysis because we hypothesized that less frequent clinic visits and associated in-person counseling might reduce continued engagement in PrEP care. We then conducted 2 sensitivity analyses for all participants and subgroups: (1) PrEP adherence at 12 months using a protective TFV-DP threshold of 700 fmol/punch or greater, which has been commonly used in other PrEP trials and implementation studies,^15,17^ and (2) continuous HIV testing using a threshold of 2 or more HIV tests completed since randomization.

## Results

Among the 495 enrolled participants (Table 1), the median (IQR) age was 33 (27-40) years, with 76 participants (15.4%) younger than 25 years. Overall, 295 (59.6%) were in serodifferent relationships. Participants had a median (IQR) number of years in school of 9 (8-12) years. Most participants were married (382 [77.2%]) and reported inconsistent condom use in the past month (405 [81.8%]). Among 330 (66.7%) female participants, 20 (6.1%) were pregnant. Following randomization, 329 participants were assigned to the intervention group (166 to blood-based HIVST and 163 to oral-fluid HIVST) and 166 to the SOC group; the demographic characteristics of participants were balanced across randomization groups.

**Table 1.** Participants’ Demographic Characteristics at Randomization, by Study Group

Characteristic	Participant, No. (%)			
	6-mo PrEP with HIVST (n = 329)^a^	Standard of care (n = 166)	6-mo PrEP with BB HIVST (n = 166)^a^	6-mo PrEP with OF HIVST (n = 163)^a^
Study population				
Men, in HIV serodifferent couple	110 (33)	55 (33)	56 (34)	54 (33)
Women, in HIV serodifferent couple	86 (26)	44 (27)	43 (26)	43 (26)
Women, singly enrolled	133 (40)	67 (40)	67 (40)	66 (41)
Age, y				
Median (IQR)	32 (27-40)	33 (28-40)	32 (27-39)	33 (28-40)
18-24	49 (15)	27 (16)	26 (16)	23 (14)
25-29	62 (19)	25 (15)	31 (19)	31 (19)
30-34	82 (25)	38 (23)	42 (25)	40 (25)
≥35	136 (41)	76 (46)	67 (40)	69 (42)
Years of education, median (IQR)	8 (8-12)	10 (8-12)	8 (8-12)	10 (8-12)
Married	256 (78)	126 (76)	127 (77)	129 (79)
Travel time to clinic				
<30 min	8 (2)	7 (4)	4 (2)	4 (3)
30 to <60 min	85 (26)	42 (25)	40 (24)	45 (28)
1 to <2 h	71 (22)	36 (22)	37 (22)	34 (21)
>2 h	165 (50)	80 (48)	85 (51)	80 (49)
Age of sexual debut, median (IQR), y^b^	17 (16-20)	18 (15-19)	17 (15-19)	18 (16-20)
Using modern method of contraception^c^^,^^d^	102 (47)	59 (53)	53 (48)	49 (45)
Currently pregnant^c^^,^^e^	14 (6)	6 (5)	7 (6)	7 (6)
Fully circumcised^f^	102 (93)	50 (91)	51 (91)	51 (94)
Sexual behaviors, past month				
Sex partners, median (IQR), No.	1 (1-1)	1 (1-1)	1 (1-1)	1 (1-1)
Frequency of sex, median (IQR), No.	8 (4-12)	8 (4-12)	8 (4-13)	8 (4-12)
Inconsistent condom use^g^	261 (79)	144 (87)	122 (74)	139 (85)
Exchanged sex for goods or money^h^	29 (9)	20 (12)	11 (7)	18 (11)
PrEP disclosure to ≥1 person^i^	62 (19)	28 (17)	27 (16)	35 (22)
Prevalence of likely depression^j^	22 (7)	5 (3)	16 (10)	6 (4)
Any social harm, past 3 mo^k^	30 (9)	10 (6)	18 (11)	12 (7)

Abbreviations: OF, oral-fluid; BB, blood-based; HIVST, HIV self-testing; PrEP, preexposure prophylaxis.

^a^ Participants randomized to these intervention arms received 6-month PrEP dispensing with interim HIVST (either oral-fluid or blood-based) with biannual clinic visits.

^b^ Missing data for 1 participant.

^c^ Outcome reported only among female participants (n = 330).

^d^ Modern methods of contraception include oral, injectable, implants, and intrauterine devices.

^e^ Missing data on baseline pregnancy status for 19 participants.

^f^ Outcome reported only among male participants (n = 165); missing data for 10 participants.

^g^ Condom use categorized as inconsistent if condoms not used during every sex act in the past month; missing data for 28 participants.

^h^ Missing data for 24 participants. Among female participants singly enrolled, 48 (24%) reported exchanging sex for money or goods in past month.

^i^ Reported PrEP disclosure to at least 1 other person besides one’s main sexual partner in serodifferent couples.

^j^ Likely depression indicates a score of 10 or greater on Patient Health Questionnaire 9-item depression scale. Data missing for 1 participant.

^k^ Social harm includes any verbal, physical, or emotional abuse by a sexual partner.

**Figure 2.**
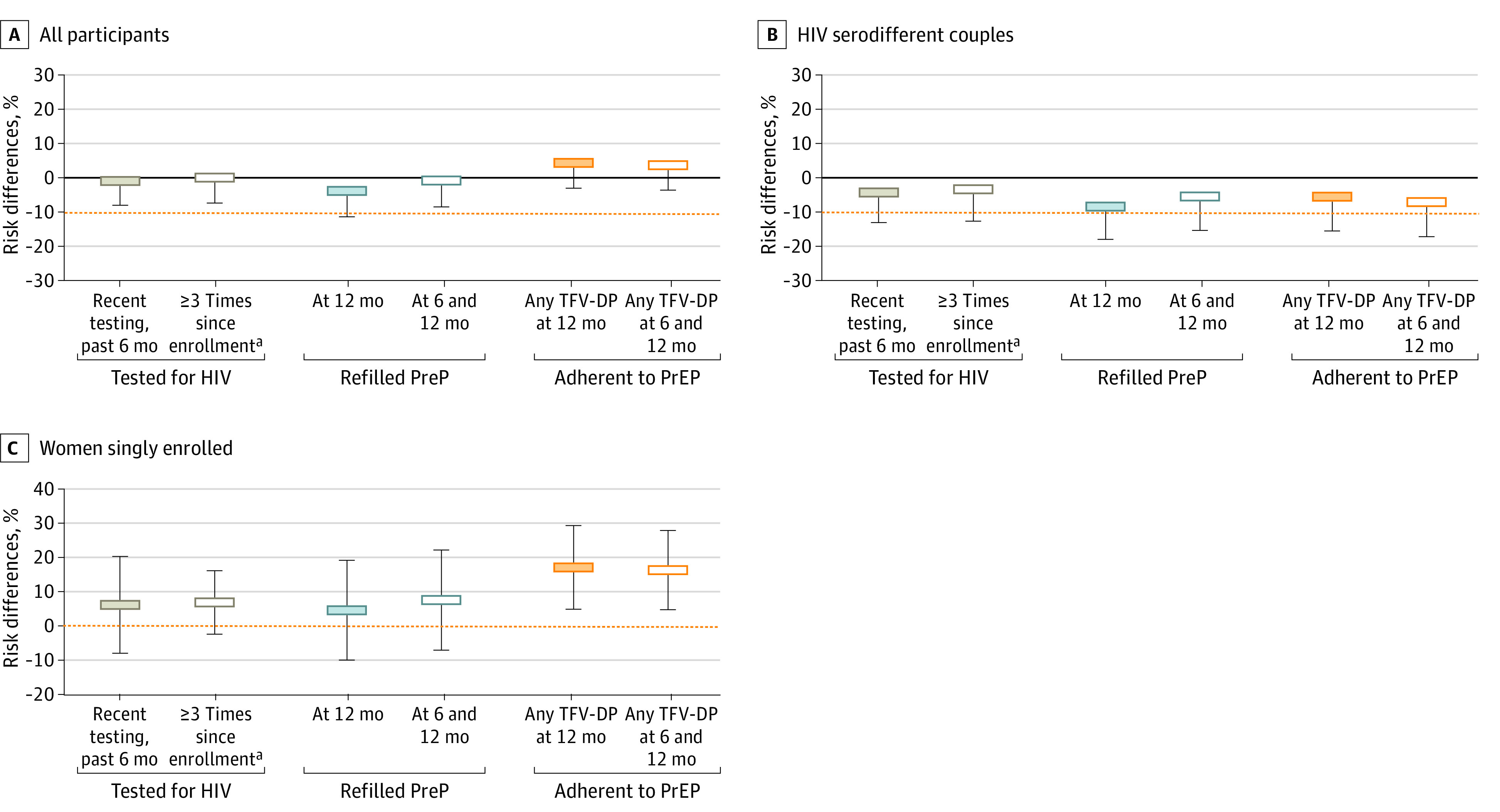
Effect of 6-Month Preexposure Prophylaxis (PrEP) Dispensing With HIV Self-testing on Study Outcomes at 12 Months Among All Participants and Participant Subgroups These risk differences compare the combined 6-month PrEP dispensing with interim HIV self-testing (either oral-fluid or blood-based) group with the standard of care 3-month PrEP dispensing group at 12 months. The orange lines indicate the marker of noninferiority (A and B) or superiority (C). TFV-DP indicates tenofovir-diphosphate. ^a^HIV testing at least 3 times determined by (1) self-report of at least 1 HIV test (any type) between enrollment and 6 months, (2) completed HIV rapid diagnostic testing at 6 months; and (3) self-report of at least 1 HIV test (any type) between 6 and 12 months.

Overall retention among those enrolled was 84.2% (417 of 495) at 6 months (intervention: 277 [84.2%]; SOC: 140 [84.3%]) and 73.0% (361 of 495) at 12 months (intervention: 241 [73.3%]; SOC: 120 [72.2%]). Retention at 12 months was 74.1% (123 of 166) in the blood-based HIVST and 72.3% (118 of 163) in the oral-fluid HIVST intervention groups. Study outcomes were generally noninferior in the intervention group compared with the SOC group (Table 2 and Figure 2). For the HIV testing outcomes, recent HIV testing was similar and noninferior in the intervention group (230 [69.9%]) compared with the SOC group (116 [69.9%]; RD, −0.33%, 1-sided 95% CI LB, −7.44%), as was continuous HIV testing (intervention: 215 [65.4%]; SOC: 107 [64.5%]; RD, 0.62%; 1-sided 95% CI LB, −6.81%). PrEP refilling at 12 months was 59.6% (196 of 329) in the intervention group compared with 62.7% (104 of 166) in the SOC group (RD, −3.25%; 95% CI LB, −10.84%), a result for which the LB of the 95% CI did not demonstrate noninferiority (ie, was inconclusive). However, PrEP refilling at both 6 and 12 months was similar and noninferior in the intervention group (186 [56.5%]) compared with the SOC group (94 [56.6%]; RD, −0.19%; 1-sided 95% CI LB, −7.93%). PrEP adherence at 12 months only and PrEP adherence at both 6 and 12 months were noninferior in the intervention group compared with the SOC group (12 months only: intervention, 151 [45.9%]; SOC, 70 [42.2%]; RD, 4.96%; 1-sided 95% CI LB, −2.46%; 6 and 12 months: intervention, 135 [41.0%]; SOC, 64 [38.6%]; RD, 4.26%; 1-sided 95% CI LB, −3.02%). When the blood-based HIVST and oral-fluid HIVST intervention were compared individually with the SOC, these findings remained largely consistent (Table 3).

**Table 2.** Effect of 6-Month PrEP Dispensing With HIVST on Study Outcomes at 12 Months Among All Participants and Participant Subgroups

Outcome	Participants, No. (%)		6-mo PrEP with HIVST vs SOC, RD (1-side 95% CI, LB)^b^
	6-mo PrEP with HIVST^a^	SOC	
**All participants**			
Total No.	329	166	NA
Returned to clinic^c^	241 (73.3)	120 (72.3)	NA
Tested for HIV			
Any in past 6 mo	230 (69.9)	116 (69.9)	−0.33% (−7.44%)
≥3 Times since enrollment	215 (65.4)	107 (64.5)	0.62% (−6.81%)
Refilled PrEP			
At 12 mo	196 (59.6)	104 (62.7)	−3.25% (−10.84%)
At 6 and 12 mo	186 (56.5)	94 (56.6)	−0.19% (−7.93%)
Adherent to PrEP			
Any TFV-DP detected at 12 mo	151 (45.9)	70 (42.2)	4.96% (−2.46%)
Any TFV-DP detected at 6 and 12 mo	135 (41.0)	64 (38.6)	4.26% (−3.02%)
**HIV serodifferent couples**			
Total No.	196	99	NA
Returned to clinic^c^	145 (74.0)	78 (78.8)	NA
Tested for HIV			
Any in past 6 mo	141 (71.9)	75 (75.8)	−3.83% (−12.63%)
≥3 Times since enrollment	133 (67.9)	70 (70.7)	−2.90% (−12.20%)
Refilled PrEP			
At 12 mo	119 (60.7)	68 (68.7)	−7.95% (−17.52%)
At 6 and 12 mo	113 (57.7)	62 (62.6)	−4.99% (−14.87%)
Adherent to PrEP			
Any TFV-DP detected at 12 mo	105 (53.6)	58 (58.6)	−5.05% (−15.08%)
Any TFV-DP detected at 6 and 12 mo	94 (48.0)	54 (54.6)	−6.63% (−16.73%)
**Women singly enrolled**			
**Outcome**	**6-mo PrEP with HIVST^a^**	**SOC**	**RD (95% CI)**
Total No.	133	67	NA
Returned to clinic^c^	96 (72.2)	42 (62.7)	NA
Tested for HIV			
Any in past 6 mo	89 (66.9)	41 (61.2)	5.72% (−8.42% to 19.87%)
≥3 Times since enrollment	82 (61.7)	37 (55.2)	6.43% (−8.06% to 20.92%)
Refilled PrEP			
At 12 mo	77 (57.9)	36 (53.7)	4.16% (−10.43% to 18.76%)
At 6 and 12 mo	73 (54.9)	32 (47.8)	7.13% (−7.52% to 21.77%)
Adherent to PrEP			
Any TFV-DP detected at 12 mo	46 (34.6)	12 (17.9)	16.68% (4.44% to 28.91%)
Any TFV-DP detected at 6 and 12 mo	41 (30.8)	10 (14.9)	15.90% (4.31% to 27.49%)

Abbreviations: HIVST, HIV self-testing; LB, lower bound; NA, not applicable; PrEP, preexposure prophylaxis; RD, risk difference; SOC, standard of care; TFV-DP, tenofovir-diphosphate.

^a^ Participants randomized to these intervention groups received 6-month PrEP dispensing with interim HIVST (either oral-fluid or blood-based) with semiannual clinic visits.

^b^ In noninferiority analyses, 1-sided 95% CIs of −10% or greater were interpreted as noninferior. RDs were measured using binomial regression models with identity links, adjusted for study population at enrollment (eg, men in HIV serodifferent couples, women in HIV serodifferent couples, and women singly enrolled).

^c^ All follow-up visits assigned as 12-month visits by study staff and that occurred prior to extensive follow-up procedures (extensive follow-up occurred when a participant had not returned for a 12-month visit by 15 months after enrollment) were included.

**Table 3.** Effect of 6-Month PrEP Dispensing and Blood-Based HIVST and 6-Month PrEP Dispensing and Oral-Fluid HIVST on Study Outcomes at 12 Months Among All Participants

Outcome	Participants, No. (%)			RD (1-sided 95% CI LB)^a^	
	6-mo PrEP with blood-based HIVST (n = 166)^b^	6-mo PrEP with oral-fluid HIVST (n = 163)^b^	SOC (n = 166)	6-mo PrEP with blood-based HIVST vs SOC	6-mo PrEP with oral-fluid HIVST vs SOC
Returned to clinic^c^	123 (74.1)	118 (72.4)	120 (72.3)	NA	NA
Tested for HIV (any in past 6 mo)	114 (68.7)	116 (71.2)	116 (69.9)	−1.63% (−9.87%)	0.88% (−7.29%)
Tested for HIV (≥3 times since enrollment)	106 (63.9)	109 (66.9)	107 (64.5)	−0.91% (−9.48%)	2.07% (−6.47%)
Refilled PrEP (at 12 mo)	100 (60.2)	96 (58.9)	104 (62.7)	−2.61% (−11.34%)	−3.96% (−12.78%)
Refilled PrEP (at 6 and 12 mo)	96 (57.8)	90 (55.2)	94 (56.6)	1.06% (−7.82%)	−1.52% (−10.49%)
Adherent (any TFV-DP detected at 12 mo)	82 (49.4)	69 (42.3)	70 (42.2)	8.45% (−0.18%)	1.36% (−6.91%)
Adherent (any TFV-DP detected at 6 and 12 mo)	74 (44.6)	61 (37.4)	64 (38.6)	7.85% (−0.61%)	0.89% (−7.24%)

Abbreviations: HIVST, HIV self-testing; LB, lower bound; NA, not applicable; PrEP, preexposure prophylaxis; RD, risk difference; SOC, standard of care; TFV-DP, tenofovir-diphosphate.

^a^ In noninferiority analyses, 1-sided 95% CIs of −10% or greater were interpreted as noninferior. RDs measured using binomial regression models with identity links, adjusted for study population at enrollment (eg, men in HIV serodifferent couples, women in HIV serodifferent couples, and women singly enrolled).

^b^ Participants randomized to these intervention groups received 6-month PrEP dispensing with interim HIVST (either oral-fluid or blood-based) with semiannual clinic visits.

^c^ All follow-up visits assigned as 12-month visits by study staff and which occurred prior to extensive follow-up procedures (extensive follow-up occurred when a participant had not returned for a 12-month visit by 15 months after enrollment) were included.

In our subgroup analyses, none of the outcomes in the intervention group were noninferior compared with the SOC group among HIV serodifferent couples, but both PrEP adherence outcomes were significantly higher in the intervention group compared with the SOC group for women singly enrolled (Table 2 and Figure 2). Specifically, PrEP adherence at 12 months among women singly enrolled was 34.6% (46 of 133) in the intervention group compared with 17.9% (12 of 67) in the SOC group (RD, 16.68%; 95% CI, 4.44%-28.91%), and PrEP adherence at both 6 and 12 months was 30.8% (41 of 133) in the intervention group compared with 14.9% (10 of 67) in the SOC group (RD, 15.90%; 95% CI, 4.31%-27.49%).

In our sensitivity analyses, PrEP adherence using a TFV-DP threshold of 700 fmol/punch or greater was superior, and continuous HIV testing using a threshold of 2 or more HIV tests was noninferior in the intervention group compared with the SOC group (eTable in Supplement 2). PrEP adherence assessed using this threshold was 32.8% (108 of 329) in the intervention group compared with 28.9% (48 of 166) in the SOC group at 12 months (RD, 6.67%; 1-sided 95% CI LB, 0.30%) and 27.7% (91 of 329) in the intervention group compared with 22.9% (38 of 166) in the SOC group at 6 and 12 months (RD, 7.53%; 1-sided 95% CI LB, 1.92%), findings that were even more pronounced in the subgroup of women singly enrolled. Continuous HIV testing at this threshold was 72.3% (238 of 329) in the intervention group compared with 71.7% (119 of 166) in the SOC group at 12 months (RD, 0.24%; 1-sided 95% CI LB, −6.75%). Over 12 months of follow-up, no study-related harms were reported, and no HIV seroconversions were observed.

## Discussion

In this study, a differentiated model of 6-month PrEP dispensing supported with interim HIVST reduced the number of health care facility visits in half without jeopardizing PrEP continuation outcomes 1 year later. Despite fewer touch points with the health system and access to associated counseling services, participants interested in PrEP continuation 1 year following initiation continued HIV testing and adhering to PrEP at similar and noninferior rates when they had semiannual vs quarterly PrEP clinic visits. Notably, for women not in known HIV serodifferent couples, this simplified model of PrEP service delivery significantly increased PrEP adherence at 1 year compared with the standard model of PrEP dispensing.

When considering scale-up of this novel PrEP service delivery model in Kenya and other settings, the potential benefits and costs of this model must be considered. Benefits include potential economic savings to the health system^18^ and more time for clinicians to serve other clients, as well as potential financial savings to clients who do not have to take days off work or spend money on transport getting to and from the health care facility.^19^ Other benefits may include clients’ feeling of empowerment and sense of control over their PrEP care and HIV risk with 6-month PrEP dispensing supported with interim HIVST, potentially resulting in better PrEP adherence. Potential opportunity costs to scaling this PrEP delivery model might include less frequent screening for PrEP adverse effects, fewer opportunities to deliver packaged interventions (eg, contraception), and limited access to associated counseling services. The prevalence of PrEP adverse effects, however, is very low, and recent studies suggest that semiannual creatinine testing is safe and would reduce health system costs.^20,21^ Additionally, women may prefer accessing HIV testing, contraception, and other associated sexual and reproductive health services in settings that are more convenient and less stigmatizing than HIV clinics (eg, private pharmacies or family planning clinics).^22,23,24,25,26^ Finally, while counseling services are a critical component of all HIV service delivery interventions, they may not be necessary on a quarterly basis for clients engaged in continued and longer-term prevention services.^27^

As newer, long-acting PrEP forms (eg, monthly vaginal rings, bimonthly injections) become increasingly available globally, differentiated models of daily oral PrEP delivery remain relevant and can have advantages over anticipated delivery models for long-acting PrEP forms. For example, oral PrEP does not require a cold chain and does not need to be delivered by a health care professional, potentially enabling more models of PrEP delivery that are community-based (eg, peer or courier delivery with mHealth interventions) and require less frequent clinic visits (eg, 6-month PrEP dispensing), which can be facilitated and supported with HIVST. Options for different PrEP modalities are needed so that clients have informed choices and can select the PrEP form and delivery model that fits their preferences, analogous to the different active pharmaceutical agents and delivery channels available for contraception.^28,29^

### Limitations

This study had limitations. First, our HIV testing outcomes relied on self-reported data subject to social desirability bias. Second, we assumed that if participants did not return for follow-up, they did not achieve the outcomes of interest. It is possible, however, that clients might have continued HIV testing and not returned for follow-up or transferred to a different clinic. Third, our retention windows for the 6- and 12-month visits were wide and thus may not have captured more nuanced ways the intervention affected clients’ PrEP use (eg, late refill visits potentially indicative of gaps in PrEP coverage or stopping and restarting PrEP use). Fourth, our study population was limited to individuals in HIV serodifferent couples and women not in known HIV serodifferent couples, so the effects of this intervention within other priority populations remains unknown. Notably, we enrolled individuals refilling PrEP 1 month following initiation; thus, our findings may not have been the same had we enrolled individuals initiating PrEP. Additionally, this trial took place at 1 research clinic, resulting in PrEP care that might not be emblematic of what participants would have otherwise received at public clinics with more limited resources in Kenya or other similar settings, potentially limiting the generalizability of our findings.

## Conclusions

In this analysis of secondary end points for a randomized noninferiority trial, we found that 6-month PrEP dispensing supported with interim HIVST reduced the number of clinic visits in half with generally noninferior PrEP continuation outcomes at 12 months compared with standard quarterly PrEP dispensing. PrEP provides high efficacy and safety for HIV prevention; continuing to optimize its delivery for those who could benefit is essential to ending the global HIV epidemic.^30^

## Notes

Supplement 1.
Trial Protocol and Statistical Analysis Plan

Supplement 2. **eTable.** Sensitivity Analyses for All Participants and Subgroups

Supplement 3.
Data Sharing Statement
