# Supplementary material for: Effect of 6-Month HIV Preexposure Prophylaxis Dispensing With Interim Self-testing on Preexposure Prophylaxis Continuation at 12 Months: A Randomized Noninferiority Trial
Source: JAMA Netw Open. 2023 Jun 15;6(6):e2318590. doi: 10.1001/jamanetworkopen.2023.18590 (PMC10273023; doi:10.1001/jamanetworkopen.2023.18590)
Supplement: Supplement 1. — Trial Protocol and Statistical Analysis Plan [file jamanetwopen-e2318590-s001.pdf]

Supplement

- eProtocol 1

ePROTOCOL 1

HIV self-testing to improve the efficiency of PrEP delivery

Version 1.7

April 18th 2019

Funding:

United States National Institutes of Health

PROTOCOL TEAM

University of Washington, Seattle, USA

Jared Baeten, MD, PhD (Co-PI)
Ruanne Barnabas, MBChB, DPhil
Deborah Donnell, PhD
Katrina Ortblad, ScD, MPH
Kenneth Mugwanya, MBChB, PhD, MS

Jomo Kenyatta University of Agriculture and Technology Nairobi, Kenya

Kenneth Ngure, PhD, MPH (Co-PI)
Elizabeth Irungu, MBChB, MPH

Kenya Medical Research Institute, Nairobi, Kenya

Nelly Rwamba Mugo, MBChB, MMed, MPH

Massachusetts General Hospital and Harvard Medical School, Boston, USA

Jessica Haberer, MD, MPH

Table of Contents

PROTOCOL SUMMARY ... 7
BACKGROUND/RATIONALE ... 8
Importance of the problem ... 8
PrEP is efficacious and safe for HIV-1 prevention. ... 8
Adherence is key to PrEP efficacy ... 8
Point-of-care HIV-1 testing is a standard component of PrEP delivery. ... 9
Self-testing offers an innovative way to test for HIV-1 ... 9
No studies have put HIV-1 self-testing and PrEP together. ... 9
INNOVATION ... 10
HIV-1 self-testing in PrEP delivery has not been formally studied ... 10
Differentiated care models of PrEP delivery are needed ... 11
Blood-based HIV-1 self-tests are new ... 11
PRELIMINARY RESULTS ... 11
PrEP is effective and safe for prevention ... 11
Costing studies and cost-effectiveness modeling ... 12
METHODS ... 13
Study Aims ... 13
Population ... 13
Study services ... 13
Eligibility ... 13
Sample size ... 14
PrEP medication ... 14
HIV-1 self-test kits ... 15
Recruitment ... 15
Randomization ... 16
Study procedures ... 16
Seroconversion ... 21
Participant retention and withdrawal ... 21
Adherence ... 21
Discontinuation of PrEP ... 21
SAFETY ... 22
Pregnancy among partner (HIV-1 uninfected) participants ... 22
HIV-testing safety ... 22
Social harm considerations for HIV-1 self-testing and PrEP ... 22
DATA AND ANALYSIS ... 23
Data collection ... 23
Qualitative data collection ... 23
Provider barriers and facilitators to HIV-1 self-testing ... 23
Outcomes ... 23
For key delivery informants ... 25
Quantitative Analysis ... 25
Qualitative Analysis ... 26
HUMAN SUBJECTS CONSIDERATIONS ... 26
Study oversight ... 26
Risks ... 26
Benefits ... 26
Care for persons identified as HIV-1 infected ... 27
Treatment for injury ... 27
Study records ... 27
Confidentiality ... 27
Dissemination Plan ... 27
Appendix I: Screening and Enrollment informed Consent – HIV uninfected ... **Error! Bookmark not defined.**
Appendix II. Screening and Enrollment informed Consent – HIV infected ... **Error! Bookmark not defined.**
Appendix III: In-depth qualitative guide ... **Error! Bookmark not defined.**
Appendix IV: Participant Information sheet with oral HIV self-test Instructions ... **Error! Bookmark not defined.**

Appendix V: Participant Information sheet with bloodbased HIV self-test Instructions........... **Error! Bookmark not defined.**
Appendix VI-Focus Group Discussion Guide .... **Error! Bookmark not defined.**

Abbreviations

3TC	Lamivudine
AIDS	Acquired Immunodeficiency Virus
AE	Adverse Event
ART	Antiretroviral Therapy
ARC	AIDS Related Complex
CDC	Centers for Disease Control and Prevention (US)
CHCT	Couples HIV testing and Counseling
DAIDS	Division of AIDS (NIH)
DALY	Disability-Adjusted Life Year
DBS	Dried Blood Spots
EC	Ethics Committee
FDA	Food and Drug Administration (US)
FTC	Emtricitabine
FTC-TP	Emtricitabine-triphosphate
HIV	Human Immunodeficiency Virus
GEE	Generalized Estimating Equations
HTTP	Hypertext Transfer Protocol (presentation of web data)
ICER	Incremental Cost-Effectiveness Ratios
IRB	Institutional Review Board
KEMRI	Kenya Medical Research Institute
NACOSTI	National Council of Science, Technology and Innovation
NIH	National Institutes of Health (US)
PrEP	Pre-exposure prophylaxis
SAS	Statistical Analysis Software
STI	Sexually transmitted infection
TDF	Tenofovir
TFV-DP	Tenofovir diphosphate
UNAIDS	Joint United Nations Program on HIV/AIDS
US	United States
UW	University of Washington
VCT	Voluntary counseling and testing
WHO	World Health Organization

PROTOCOL SUMMARY

Maximizing access and minimizing costs of delivery are key challenges for optimizing the public health impact of pre-exposure prophylaxis (PrEP) for HIV-1 prevention, particularly for resource-constrained settings. PrEP is highly effective and safe when taken as prescribed, and demonstration studies are showing how PrEP can be delivered in clinical settings. In Africa, PrEP will be added to an already-burdened health infrastructure and the ability of public health systems to afford PrEP will necessitate making its delivery cost-effective and time-efficient. PrEP delivery programs will need to be cost-sensitive to staffing needs (e.g., frequent clinic visits); moreover, patients may not continue PrEP if their costs (e.g., travel to / waiting in clinics) are high. HIV-1 testing is central to PrEP: testing must occur prior to initiation and ongoing HIV-1 testing is essential for delivery. Like PrEP, HIV-1 self-testing is a recent innovation and its opportunities to improve HIV-1 prevention have not been fully realized. We hypothesize that HIV-1 self-testing can streamline PrEP delivery – through decreasing the frequency of PrEP clinic visits by having self-tests at home replace clinic-based testing. *Both oral fluid and new finger stick blood-based HIV-1 self-tests could be used, and these two modalities might have different costs or preferences.*

In May 2017, Kenya announced national scale-up of PrEP for persons at risk for HIV-1, prioritizing HIV-1 serodiscordant couples, women at risk, and other priority groups, and also the prioritization of HIV-1 self-testing in the country. This project proposes to address key access and cost of delivery challenges for PrEP by integrating the new modality of HIV-1 self-testing, with the following Aims:

Aim 1: **In a randomized trial, we will test the use of HIV-1 self-testing to decrease the frequency and burden of clinic visits for PrEP while resulting in equivalent adherence and testing.**

Hypothesis: Guidelines recommend HIV-1 testing quarterly on PrEP; we propose HIV-1 self-testing at home could alternate (e.g., Months 3, 9) with clinic-based testing (e.g., Months 6, 12), eliminating half of clinic visits and saving staffing and patient costs. Reducing clinic contact frequency will not result in reductions in PrEP adherence or completion of HIV-1 testing, overall or in subgroups.

Approach: **Design:** We will conduct a randomized trial using a non-inferiority design among 495 women and men at risk for HIV-1 in Kenya initiating PrEP. **Population:** We will enroll men (n=165) and women (n=165) who are HIV-1 uninfected partners in HIV-1 serodiscordant couples and women at risk of HIV-1 (n=165), populations prioritized for PrEP delivery in Kenya and more generally in Africa. **Intervention:** Approximately one month after PrEP initiation, participants will be randomly assigned in a 2:1 fashion to either: six-monthly clinic visits with either oral fluid-based or blood-based HIV-1 self-testing at home for quarters between clinic visits (self-testing arm) or quarterly clinic visits with in-clinic finger stick blood-based rapid HIV-1 testing (standard of care arm). **Follow-up:** PrEP refills will occur at clinic visits – quarterly (standard of care) and 6-monthly (self-testing), outcomes will be measured at Months 6 and 12. **Outcomes:** PrEP adherence (defined by PrEP quantity in dried blood spots and persistence in refilling PrEP), HIV-1 testing, and safety (including side effects and social harm).

Aim 2: **We will conduct mixed-methods work to understand user and provider experiences, preferences, barriers, and facilitators related to HIV-1 self-testing.**

Hypothesis: HIV-1 self-testing will appeal to patients, because of greater self-efficacy and reduced opportunity costs, and providers, for reduced workload. Blood-based tests *may inspire* greater confidence than oral fluid tests. Gender and partner involvement may influence acceptability of HIV-1 self-testing.

Approach: Triangulating data from structured survey and qualitative interviews, we will assess patient and provider perceived benefits of and concerns about HIV-1 self-testing in the context of PrEP.

Aim 3: **We will assess costs and cost-effectiveness of HIV-1 self-testing to optimize PrEP delivery.**

Hypothesis: HIV-1 self-testing will decrease the cost of PrEP delivery and improve PrEP cost-effectiveness.

Approach: Using activity-based micro-costing data and outcome information from Aim 1, we will define the costs and model the cost-effectiveness of HIV-1 self-testing for optimized PrEP delivery.

Strategies to decrease the frequency of PrEP follow-up visits would improve its cost-effectiveness, reach, and impact. Combining self-testing and PrEP brings together two cutting-edge interventions, and the simple HIV-1 self-testing strategy in this application could cut the number of PrEP follow-up visits in half.

BACKGROUND/RATIONALE

Importance of the problem

More than two million persons become newly infected with HIV-1 each year, the majority in sub-Saharan Africa [1]. In Kenya, more than 1.4 million people are living with HIV-1 [2], making it the country with the fourth greatest number of persons living with HIV-1. The past five years have witnessed major strides in the development of highly-effective HIV-1 prevention interventions, particularly ones using antiretroviral medications: antiretroviral therapy (ART) for HIV-1 infected persons to decrease infectiousness and pre-exposure prophylaxis (PrEP) for uninfected persons to prevent acquisition. Novel strategies to successfully and efficiently deliver these strategies are needed to achieve maximum impact among populations at the highest risk of HIV-1.

PrEP is an effective and recommended strategy for HIV-1 prevention

PrEP is efficacious and safe for HIV-1 prevention.

PrEP has been demonstrated to be efficacious and safe for reducing HIV-1 risk among men who have sex with men [3], heterosexual men and women [4, 5], and injection drug users [6] in diverse geographic settings. In 2012, the US Food and Drug Administration approved combination tenofovir disoproxil fumarate/emtricitabine (TDF/FTC) as the first medication with a label indication for HIV-1 prevention in adults [7] – an action followed by drug regulatory authorities in a number of other countries including Kenya (in December 2015). In 2015, the World Health Organization issued guidance recommending PrEP as an additional prevention option for all persons at high risk for acquiring HIV-1 [8]. The 2016 Kenyan ART guideline have incorporated Tenofovir/Emtricitabine (TDF/FTC) as the preferred regimen with Tenofovir/Lamivudine (TDF/3TC) and Tenofovir (TDF) alone as recommended options for HIV-1 pre-exposure prophylaxis [58].

Adherence is key to PrEP efficacy.

Like for ART, adherence is essential for PrEP efficacy. PrEP clinical trials had a wide range of results for estimates of PrEP's efficacy for HIV-1 protection – explained by the degree to which the trial populations were adherent to PrEP [9]. Secondary analyses from clinical trials and demonstration studies have shown that PrEP is clearly efficacious when taken as prescribed. At the individual level, HIV-1 protection is on the order of 90-100% in both men who have sex with men and heterosexual populations when PrEP adherence was high, as measured by the presence and quantity of PrEP in blood samples [4, 10, 11]. PrEP adherence and HIV-1 prevention effectiveness have been higher in open-label demonstration projects among HIV-1 serodiscordant couples, men who have sex with men, and young women at risk for HIV-1 than in prior clinical trials [12-14], which has been hypothesized to be a result of offering a strategy with demonstrated safety and effectiveness, and without a placebo. In those PrEP demonstration studies, HIV-1 incidence has been low and visits were generally quarterly and brief, suggesting that many who initiate PrEP in the context of known safety and efficacy may not need frequent or intensive follow-up to achieve high adherence.

Strategies to effectively and efficiently deliver PrEP are needed

PrEP delivery can be expensive, in terms of medication costs, staffing time, laboratory testing, and patient opportunity costs. Multiple analyses from high-income settings have argued that, while PrEP is cost-effective when delivered to high-risk persons, it is still a costly intervention [15-17]. For developing country settings, the results are similar – even when taking into account lower cost inputs such as generic or discounted medication pricing, lower staff salary costs, and more truncated laboratory testing recommended by WHO and country policies [16, 18]. Thus, costs will be a barrier to PrEP delivery for impact, in all settings.

In costing analyses we have conducted in East Africa, we estimated that adding PrEP to routine public health services using Ministry of Health personnel, drug, and laboratory costs would cost ~US$100 annually per person. Notably, in those models, the greatest proportion of the total costs was not medication costs but was instead

personnel (39%) [19]. That finding emphasizes the need for efficiency in PrEP delivery, particularly so given that qualified medical staff in Kenya and similar settings are often highly over-stretched, because of competing priorities in overburdened health systems. Our modeling did not take into account patient costs related to PrEP; however, we have learned through providing PrEP in studies to over 5000 individuals over the past 10 years that travel and time away from work and costs for getting to PrEP clinic visits can be a challenge for persons taking PrEP. Efficient strategies to deliver PrEP could reduce costs, potentially improving patient engagement and allow services to be available to a larger number of people as a result – and this kind of approach would be applicable in a variety of settings worldwide.

<u>HIV-1 testing is central to PrEP; HIV-1 self-testing is a novel approach that could streamline PrEP delivery</u>

Point-of-care HIV-1 testing is a standard component of PrEP delivery.

PrEP requires regular HIV-1 testing – at the time of PrEP initiation and then in an ongoing basis. HIV-1 testing is necessary to reduce the risk of antiretroviral resistance if HIV-1 infection is present prior to PrEP, in which case PrEP will not be started, or occurs while prescribed PrEP, in which case it will be discontinued. PrEP clinical trials were carried out in the context of monthly HIV-1 testing, generally using point-of-care third-generation antibody-based tests, conducted at the clinical trial sites. Although this frequent approach to HIV-1 testing was important for the clinical trials, which were the first evaluations of PrEP use, monthly testing would be logistically impossible for routine public health settings because of excessive costs and burden to PrEP takers and providers. Thus, initial guidance about PrEP delivery from the US FDA and CDC recommended testing every three months, and demonstration projects of PrEP distribution conducted quarterly testing [13, 14, 20]. The safety of this approach has been borne out in analyses of HIV-1 acquisition among persons receiving PrEP, which have repeatedly demonstrated that the risk of HIV-1 during PrEP use is greatest at the start of PrEP (i.e., unrecognized acute infection coincident with PrEP initiation), that HIV-1 acquisition after PrEP initiation is rare except for persons not taking PrEP, and that resistance risk is thus also rare during PrEP follow-up (as either persons take PrEP and do not get HIV-1 or don't take PrEP and thus cannot select for resistance of they acquire HIV-1) [3, 4, 21, 22]. WHO recommends quarterly testing as the global standard in an effort to balance costs, safety, and burden to PrEP takers and providers [8], and US and global guidelines do not restrict either the assay (point-of-care versus laboratory, antibody- vs. antigen/RNA-based) or the location (in-clinic versus laboratory versus home) of HIV-1 testing for persons receiving PrEP follow-up. Nevertheless, to our knowledge, no programs distributing PrEP have attempted to move HIV-1 testing out of clinical settings and into the home with self-testing, and evidence is needed to motivate policy change for this opportunity. As PrEP expands into public health clinical settings there are new opportunities for easier models of HIV-1 testing.

Self-testing offers an innovative way to test for HIV-1

In 2012, the US FDA approved use of the OraQuick In-Home HIV Test as the first self-administered test for HIV-1, opening a new opportunity for evaluating HIV-1 testing directly under the control of an individual [23]. Several other self-testing assays in addition to OraQuick are under development or available. Self-testing offers strong advantages: privacy and convenience, and these may appeal to both patients and providers. HIV-1 self-testing has been proposed as a strategy to increase both first-time HIV-1 testing (i.e., as a general population screening tool) and repeat HIV-1 testing (i.e., for persons with ongoing HIV-1 risk), both of which could be extremely valuable in high-prevalence settings. A study conducted in Malawi demonstrated high use and accuracy: only 8% of subjects chose not to test and self-testing results were 99% concordant with rapid finger-stick tests collected in parallel [24]. In Kenya, a recent population based survey found 74% reported willingness to use HIV-1 self-test kits for first-time HIV-1 testing [2]. Ongoing projects, some at very large scale [25, 26], are evaluating wide-scale distribution of HIV-1 self-tests in Africa. WHO now recommends HIV-1 self-testing as an additional testing option for individuals and countries [27].

No studies have put HIV-1 self-testing and PrEP together.

Evaluating HIV-1 self-testing within a PrEP context has not been evaluated, although our pilot work (detailed below) suggested both feasibility and acceptability. In the present study, we propose that HIV-1 self-testing can reduce facility-based periodical testing and clinics visits among persons receiving PrEP and can do so safely and without detrimentally affecting adherence. Our proposed model has several potential advantages. First, self-testing could provide an efficient method that achieves the objectives of achieving high adherence to PrEP and ensuring HIV-1 testing is done without increasing the delivery costs and participant burden, compared to standard of care. Second, both PrEP and self-testing are new and seeing how they fit together is an incredible new opportunity. Third, efficiency of delivery could be appealing not only to the policy makers (reduced cost) and health providers (reduced work load) but also to the PrEP users (reduced clinic visits).

Summary: unanswered questions and central hypothesis

For persons taking PrEP for HIV-1 prevention, regular HIV-1 testing at fixed-site clinics has been the standard. We hypothesize that HIV-1 self-testing can replace some of the periodic follow-up HIV-1 testing otherwise done at a facility for persons receiving PrEP, reducing clinic and patient burdens. As detailed below, the proposed work will build on and extend our prior work in PrEP and HIV-1 self-testing, by proposing a novel model of delivering PrEP through fewer clinic visits which has the potential to revolutionize PrEP delivery.

The Government of Kenya has proposed a visionary 20-year plan for evidence-based HIV-1 prevention, including prioritizing PrEP and encouraging use of HIV-1 self-testing. In Kenya, the Ministry of Health developed the Kenya HIV Prevention Revolution Road Map: Count Down to 2030, a national plan to drive new HIV-1 infections towards zero between 2013 and 2030 [28]. The approach is based on key concepts in combination prevention: recognition of a heterogeneous epidemic, prioritization of sub-populations for prevention (HIV-1 serodiscordant couples, sex workers, men who have sex with men, and women at risk), and delivery of evidence-based prevention. Kenya was one of the first countries to implement point-of-care testing for HIV-1 [29, 30], and the Ministry of Health has supported research for self-testing. In May 2017, Kenya formally launched PrEP and HIV-1 self-testing as part of this plan (http://www.nation.co.ke/news/Govt-launches-two-approaches-to-fight-HIV-Aids/1056-3914614-8p6ubc/). Our proposed work aligns tightly with the Kenya HIV Prevention Revolution Road Map and our work will be supported by the Kenya Ministry of Health (see letter of support).

INNOVATION

PrEP is a potent and safe HIV-1 prevention strategy. To maximize impact, optimal approaches are now needed for delivering PrEP effectively and efficiently, particularly for resource-constrained settings. In our recent PrEP demonstration project among HIV-1 serodiscordant couples in East Africa, HIV-1 transmission was virtually eliminated [14]. In that study, we delivered PrEP with quarterly clinic-based visits, and we piloted HIV-1 self-testing at home between quarterly visits, finding that self-testing was acceptable and feasible in the setting. We now propose to extend that work and simplify PrEP delivery further with HIV-1 self-testing as a strategy to reduce the frequency of clinic visits (from quarterly to six-monthly), which we will test through a randomized trial. This approach is highly innovative, in four key ways: integration of HIV-1 self-testing into PrEP, understanding for whom the proposed approach works and does not work (as a model of differentiated care for HIV-1 prevention [31]), testing of blood-based HIV-1 self-tests, and support by the government of Kenya, which makes the probability of impact high.

HIV-1 self-testing in PrEP delivery has not been formally studied

PrEP is a biomedical intervention, and its evaluation in clinical trials and demonstration settings has been clinic based. However, with greater deployment of PrEP in many settings, strategies to simplify its delivery have been a priority. We hypothesize that HIV-1 self-testing can successfully be used to conduct the HIV-1 testing that is a necessary part of PrEP delivery, but which can be done by PrEP patients instead of clinic-based staff. Reducing the frequency of follow-up clinic visits has the potential to reduce staffing costs associated with frequent visits and client-related opportunity costs such travel to the clinic and waiting time which could increase client persistence with PrEP use. While many studies are using HIV-1 self-testing to link people to care and prevention, self-testing use among those taking PrEP has not been done to our knowledge, and has potential to further efficient strategies for PrEP provision.

Differentiated care models of PrEP delivery are needed

In HIV-1 treatment settings, simplified approaches to follow-up care (e.g., 6-monthly visits for those with stable viral suppression) allow human and financial resources to be directed to those individuals needing more attention, a concept known as differentiated care. For PrEP, differentiated care models have not been developed yet, and thus a "one size fits all" approach is being done. It is very likely that many patients receiving PrEP, like those on ART, can achieve high adherence without frequent follow-up. We will conduct subgroup analyses (in Aim 1) and use mixed-methods behavioral science (in Aim 2) to explore how HIV-1 self-testing fits into PrEP provision and for whom this simplified HIV-1 testing and follow-up approach to PrEP works well and for whom it does not work well, including assessment of gender and partner knowledge of PrEP use, as a first approach to defining a differentiated care model for PrEP.

Blood-based HIV-1 self-tests are new

The OraQuick oral fluid-based HIV-1 self-test is an established, FDA-approved product. Blood-based HIV-1 tests are new and have not been tested nearly as widely. However, blood tests are commonly used in clinics for HIV-1 testing, and blood-based testing could offer greater confidence in results to at least some patients (and providers). In Aim 2, we will directly compare acceptability, usability, and confidence for oral fluid versus blood for HIV-1 self-testing.

PRELIMINARY RESULTS

We have conducted randomized trials, behavioral science, implementation science, and mathematical modeling to define the efficacy, acceptability, and cost-effectiveness of PrEP for HIV-1 prevention.

PrEP is effective and safe for prevention

We conducted the Partners PrEP Study, the randomized clinical trial that demonstrated the efficacy of PrEP for HIV-1 prevention in heterosexual populations [4, 32]. Secondary analyses from that trial confirmed that PrEP provided significant protection including in key subgroups: both men and women [33], couples with high-risk characteristics (e.g., those practicing unprotected sex, couples in which the HIV-1 infected partner had a high plasma viral load, those with sexually transmitted infections) [33], and in women using contraception [34]. We also conducted analyses to demonstrate limited selection of antiretroviral resistance [21], safety in women using contraception or who become pregnant [35, 36], and limited renal toxicity [37-39]. Our work has been central to the development of PrEP guidance for populations worldwide [40, 41].

We conducted a large-scale demonstration of integrated ART and PrEP that showed near-elimination of HIV-1 transmission. We conducted the Partners Demonstration Project, an implementation science project delivering PrEP among 1013 HIV-1 serodiscordant couples who attended four HIV-1 research centers in East Africa. The primary goal was to demonstrate a model of PrEP delivery; ART was promoted for all couples for clinical and prevention benefits, and PrEP offered until 6 months after ART initiation by the infected partner, permitting time to achieve viral suppression, a pragmatic strategy we called "PrEP as a bridge to ART", which has directly influenced Kenya's PrEP guidelines for couples; in this study, the majority of couples used PrEP for at least 12 months, because many HIV-1 infected partners delayed ART initiation for at least 3 months or more. We found high adherence (85% of blood samples had PrEP detected) and near-elimination of HIV-1 transmission: only 4 infections observed (all among persons not using PrEP)

Figure 1. Near-elimination of HIV-1 transmission using an integrated delivery strategy for ART and PrEP: the Partners Demonstration Project

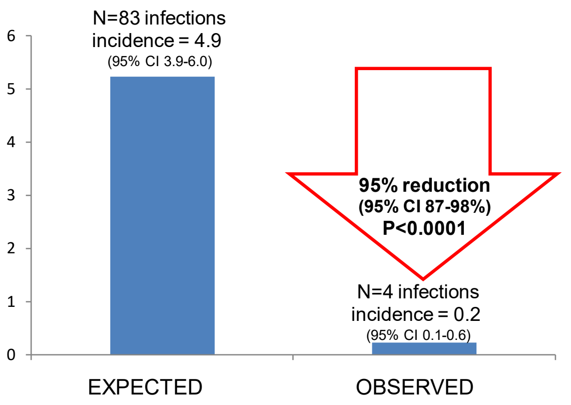

compared to 83 in a simulated counterfactual cohort (**Figure 1**).

Pilot study: *HIV-1 self-testing is highly acceptable in the context of PrEP.* Within the Partners Demonstration Project, we completed a pilot evaluation of HIV-1 self-testing at the Thika site [42]. OraQuick HIV-1 oral fluid self-test kits were provided for use, at home, in the two-month interval between scheduled quarterly clinic visits. We found 222 of 226 (98%) persons on PrEP offered self-testing accepted and nearly all (96.8%) reported using the self-testing kit was easy. Many reported that HIV-1 self-testing was empowering (**Table 1**); there was a range of opinions about oral fluid versus blood tests for HIV-1, with some participants voicing greater confidence in blood (which we also heard anecdotally from providers working as part of that pilot). Across all visits, a total of 1225 HIV-1 self-testing kits (95.6% of 1282 dispensed) were reported to have been used, 253 (98.1%) and 972 (94.9%) among HIV-1 uninfected women and men, respectively. A total of 17 calls were made to the 24-hour helpline in relation to challenges in performing or interpreting the test results; one was a positive test, not confirmed on follow-up testing, and no participants seroconverted to HIV-1. Other studies in Kenya have demonstrated that HIV-1 self-testing at home is acceptable for women at risk of HIV-1, including women at risk not in known HIV-1 serodiscordant couples [43, 44]

**Table 1. Experiences with HIV-1 self-testing and PrEP: pilot study**
Reduced anxiety<br>• "Every day, every time thinking, "How will it be when I go back there [clinic]? When you test yourself, you know your status, you relax"<br>Testing alone versus with others:<br>• "Sometimes I call my husband. Like the last time I tested, I called my husband and told him "Come you see mine is okay until now."<br>• "Like me, I hide myself… [Participants laugh] I go to the bedroom."<br>Preferences of HIV-1 self-tests:<br>• "You know … to many people…they know blood is the accurate one. But you know this [oral self-test kit] doesn't have any blood.<br>• "You know sometimes for blood draw it can happen you [referring to himself] prick yourself wrongly. And you have nothing to prevent germs from getting in… But this one [oral kit] is easy…."

Costing studies and cost-effectiveness modeling.

We have extensive experience with cost and cost-effectiveness studies to understand the deliverability of prevention interventions. Within the Partners Demonstration Project, we conducted micro-costing and time and motion analyses in Uganda [19]. The cost of PrEP (and ART) was assessed, with and without research components. Then, using Ministry of Health data, the costs within a government program were estimated. We parameterized an HIV-1 transmission model to estimate the health and economic impacts of the intervention, with incremental cost-effectiveness ratios (ICERs) per HIV-1 infection and disability-adjusted life year (DALY) averted calculated over 10 years. We found that the annual cost of PrEP and ART delivery for couples would be $453 in the government setting, with $92 due to PrEP. Over 10 years, a program of PrEP and ART for couples was projected to cost $1340 per infection averted. By Uganda's gross domestic product per capita of $1681, this intervention thus is cost-effective [19]. At the Thika, Kenya site, we recently completed a similar costing exercise (Irungu, Ngure, Baeten, et al., data presented at R4P conference, October 2016 and submitted). Using Kenya Ministry of Health personnel, drug, and laboratory costs, we estimated that the incremental cost of delivering PrEP in Kenya to be $110 per couple per year, with a total cost of $544 per couple per year for ART plus PrEP. The largest cost was due to personnel (39%) – emphasizing the importance of efficiencies – followed by medication (34%).

METHODS

Taking PrEP to scale will require simplifying models for delivery, for cost savings and patient preference. We have assembled a multidisciplinary team to test a simplifying strategy for PrEP delivery. We hypothesize that alternating home HIV-1 self-testing with clinic based HIV-1 testing – with resulting reductions in clinic visits from quarterly to semiannually – will translate into cost savings for PrEP programs as well as patient related opportunity costs, without reducing PrEP adherence, and will be feasible, acceptable, preferred, and safe.

Study Aims

Aim 1: **In a randomized trial, we will test the use of HIV-1 self-testing to decrease the frequency and burden of clinic visits for PrEP while resulting in equivalent adherence and testing.**

Hypothesis. Global guidelines recommend HIV-1 testing quarterly for persons on PrEP; we propose that HIV-1 self-testing could alternate (e.g., Months 3, 9) with clinic-based testing (e.g., Months 6, 12) to cut the frequency of clinic visits in half, saving staffing and patient costs, without reducing PrEP adherence or persistence.

Aim 2: **We will conduct mixed-methods work to understand user and provider experiences, preferences, barriers, and facilitators related to HIV-1 self-testing.**

Hypothesis. HIV-1 self-testing will appeal to patients, because of greater self-efficacy and reduced opportunity costs, and providers, for reduced workload. Blood-based tests may inspire greater confidence. Understanding for whom self-testing "works" will help define differentiated care models for PrEP delivery.

Aim 3: **Assess the cost and cost-effectiveness of HIV-1 self-testing to optimize PrEP delivery.**

Hypothesis. HIV-1 self-testing will decrease the cost of PrEP delivery and improve PrEP cost-effectiveness, allowing for greater efficiency, reach, and impact.

Population

At the Thika clinic, we will recruit women and men in HIV-1 serodiscordant relationships (n=165 HIV-1 uninfected women and n=165 HIV-1 uninfected men) and HIV-1 uninfected women at risk (n=165) who have recently initiated PrEP for this project. We will aim to have equal enrollment for the three study groups, but will over-enroll HIV-1 uninifected women at risk if we have difficulties or delays in enrolling men or women in HIV-1 serodiscordant relationships, whom historically have been more difficult to enroll.

Study services

<u>Standard HIV-1 testing arm</u>
Participants will receive baseline and quarterly HIV-1 counseling, condoms, risk reduction counseling, and syndromic management of sexually transmitted infections according to local guidelines.

<u>HIV-1 self-testing arm</u>
Participants will receive baseline and semiannual HIV-1 counseling, condoms, risk reduction counseling, and syndromic management of sexually transmitted infections according to local guidelines.

<u>Both study arms</u>
To ensure as real world assessment of uptake and sustained use of PrEP as possible, tracing of participants will occur only for follow-up of safety issues and for HIV-1 assessment, but not for completion of routine visits and PrEP refills.

Eligibility

Inclusion

- Age ≥18 years HIV-1 uninfected based on negative HIV-1 rapid testing
- Not currently enrolled in an HIV-1 prevention clinical trial
- Taking PrEP and planning to continue
- Willing to be randomized to either clinic based HIV-1 testing or HIV-1 self testing
- Note: Women who are pregnant at screening/enrollment are still eligible

For the HIV-1 serodiscordant couples, HIV-1 infected members of the couple will be enrolled for a single visit at baseline, if:

- Age ≥18
- Able and willing to provide written informed consent

Exclusion

- Unable to provide written informed consent
- Contraindication to use TDF/FTC/3TC

Sample size

A total of 825 individuals will be recruited: 165 HIV-1 uninfected men and their partners in HIV-1 serodiscordant relationships, 165 HIV-1 uninfected women and their partners in HIV-1 serodiscordant relationships, and 165 HIV-1 uninfected women at-risk for HIV-1 who are not in disclosed HIV-1 serodiscordant partnerships. If we have difficulties enrolling HIV-1 uninifected men or women in serodiscordant relationships, we will plan on over-enrolling HIV-1 uninifected women at-risk for HIV-1 who are not in disclosed serodiscordant relatinships to ensure we have sufficient statistical power to determine interventional effectiveness.

Table 2. Sample distribution

**Cohort**	**Number**	**HIV-1 Infected Partners**	**Number**	
Women in HIV-1 serodiscordant relationships	165	Men	165	
Men in HIV-1 serodiscordant relationships	165	Women	165	
Women	165	n/a	n/a	**TOTAL**
	**495**		**330**	**825**

PrEP medication

Tenofovir disoproxil fumarate (or TDF, 9-[(R)-2-[[bis [[(isopropoxycarbonyl) oxy] methoxy] phosphinyl] methoxy] propyl] adenine fumarate), emtricitabine (or FTC, 5-fluoro-1-(2R,5S)-[2-(hydroxymethyl)-1,3-oxatholan-5-yl]cytosine), and lamivudine (or 3TC, 2',3'-dideoxy-3'-thiacytidine 4-Amino- 1-[(2R,5S)- 2-(hydroxymethyl)- 1,3-oxathiolan-5-yl]- 1,2-dihydropyrimidin- 2-one) are reverse transcriptase inhibitors that have been approved for the treatment of HIV-1 infection in humans in Kenya and the United States. A fixed-dose, oral co-formulation of FTC/TDF (Truvada®) has also been approved for HIV-1 prevention in Kenya and the United States. The World Health Organization recommends TDF-containing medications as PrEP, which includes TDF combined with FTC as well as potentially TDF alone and TDF combined with lamivudine (or 3TC, a medication closely related to FTC). Any TDF-containing medications that align with WHO and Kenya national guidelines for PrEP will be used in this study. PrEP will be prescribed for once-daily use. Study medication will be provided by the Kenya Ministry of Health.

The study drug will be stored in accordance with the drug manufacturer's recommendations. The pharmacy and storage facility will have locked, climate-controlled environments, with controlled humidity and temperature to remain within limits allowed by the manufacturer for drug storage. Dispensing will be sufficient to last until the

next visit – thus, at enrollment month, a three month and six monthly supply depending on the study arm.

Counseling on the medications being used, their side effect profiles, how to take the study medication, what to do if side effects are experienced, and the importance of not sharing study medication to optimize potential efficacy and to reduce the chances of developing resistance through suboptimal HIV-1 suppression if study medication is shared with others.

HIV-1 self-test kits

The **OraQuick In-Home HIV Test (Figure 2)** is the first FDA approved test (2012) that uses oral fluid to test for antibodies to HIV-1 and HIV-2. The OraQuick In-Home HIV Test is a qualitative test that gives visually read results: preliminary positive, negative, or test not working (invalid) in about 20 minutes and its ease of use makes it ideal as an in-home test kit. The OraQuick oral fluid test has a sensitivity 91.7 percent and a specificity of 99.9 percent and is categorized as a "screening" test, therefore a second test to confirm the results is recommended.

Figure 2. The OraQuick In-Home HIV Test

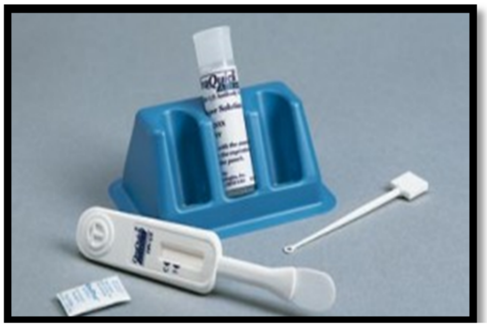

The **AtomoRapid™ HIV (1&2) Test (Figure 3,** (http://atomodiagnostics.com/ products/atomorapid-hiv/) is an integrated HIV test of blood for the presence of antibodies to HIV-1 and HIV-2. It uses a contact-activated auto-retracting safety lancet, which safeguards against needlestick injuries and cross contamination (all material and waste are within the kit itself). The all-in-one device puts the end user first and makes it easy to test. AtomoRapid's interlocking features ensure each user step is performed in the correct sequence, which helps to reduce user errors. Additionally, its blood collection and delivery is controlled which simplifies test procedures, improves blood volume accuracy and delivers blood to the correct location on the test strip. The AtomoRapid™ HIV (1&2) Test has demonstrated sensitivity 99.8% and a specificity of 100% in laboratory tests conducted by the Institute of Tropical Medicine, Belgium and the German Red Cross. Pilot studies of the AtomoRapid™ HIV (1&2) Test have been done in both Kenya and South Africa. The retail price of the AtomoRapid™ HIV (1&2) Test in Kenya is anticipated to be about 20-40% less than of the current price of the OraQuick In-Home HIV Test in Kenya (personal communication, Atomo Diagnostics).

Figure 3. The AtomoRapid™ HIV (1&2) Test

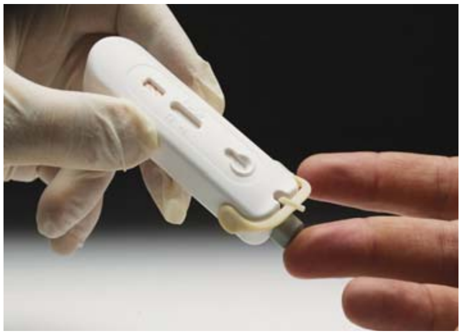

If there are any issues or interruptions with the procurement of AtomoRapid HIV self-testing kits in country, we will procure and train participants on the use of other HIV self-testing kits that have been prequalified by the WHO, an official observer of the International Medical Device Regulators Forum. We will ensure that all HIV self-testing kits utilized by participants in this study will have a greater than 95% sensitivity and specificity. A list of applicable HIV self-testing kits is available in the UNITAID's HIV Rapid Diagnostic Tests for Self-Testing, 4th Edition. [45]

Recruitment

The Thika site has established local recruitment and screening methods that operationalize protocol-specified requirements for eligibility determination in a manner that is tailored to and most efficient for the local study setting and target study population.

Thika is an urban center, about 40 km outside of Nairobi, and has a large peri-urban and rural population base surrounding it; over the past decade, the Thika site team established a multi-disciplinary site, focused on HIV-1 and sexually transmitted disease prevention research (more than two dozen projects) and on provision of clinical care

(HIV-1 testing, HIV-1 comprehensive care, HIV-1 prevention services). Our experienced community outreach team at the Thika site has established successful recruitment strategies, including collaborating with existing HIV-1 testing centers and community-based mobilization for couples and women to engage in HIV-1 prevention. The Thika clinic is a center of excellence for PrEP in Kenya and is leading training of other clinics as part of Kenya national scale-up.

Recruitment strategies will include partnering with existing voluntary counseling and testing (VCT) centers and outreach workers, public promotion of couples VCT by well-known figures and community organizations such as churches, and community mobilization around couples and women's VCT promotion (e.g., around Valentine's Day). Recruitment materials will educate couples and women about PrEP. Individuals on PrEP referred to the Thika clinic or those initiating PrEP at the Thika clinic will then be recruited to this study approximately one month after PrEP initiation.

Screening and enrollment may occur on the same day or may be split across days, depending on the preferences of the potential participant. Informed consent for study participation and enrollment in the study may proceed on the same day when eligibility is determined. For couples, HIV-1 infected partners will provide separate informed consent for a single visit but will not have longitudinal trial follow-up.

Randomization

Randomization will be done in variable-sized blocks using opaque envelopes opened at the time of randomization. Randomization will occur in a 2:1 fashion to alternating HIV-1 testing at home and in-clinic testing, or standard of care (HIV-1 testing at in-clinic follow-up visits every 3 months). Those randomized to the self-testing arm will then be further randomized in a 1:1 fashion to either blood-based or oral fluid-based self-testing kits. Randomization will be stratified by group (HIV-1 uninfected men in serodiscordant couples, HIV-1 uninfected women in serodiscordant couples, and HIV-1 uninfected women at risk). Randomization will be done at the enrollment visit which will occur approximately one month after the participants have begun taking PrEP.

Study procedures

Specific study procedures will take place at screening and enrollment, and then quarterly for the standard testing arm and biannually for the HIV self-testing arm, for up to 12 months.

At screening, eligibility information will be collected. Subjects who meet the eligibility criteria will then be enrolled in the study.

Demographic and behavioral information will be collected at enrollment. As part of PrEP intiation and continuation, several standard of care laboratory tests will be completed including HIV-1 testing, serum creatinine, and a urine pregnancy test. For this study, additional information will be collected on medical history, physical exam findings, and STI syndromic assessment and additional testing will be done as part of standard of care PrEP follow-up. Other laboratory tests will be conducted to measure adherence to PrEP medication by testing drug levels in the collected blood specimen (dried blood spots, blood hemoglobin, and blood plasma).

HIV-1 positive participants will also be assessed at baseline. Assessment will include demographic and sexual-behavior information, ART adherence data, and a brief medical history. Laboratory procedures carried out on HIV positive participants will include standard HIV-1 testing, CD4 count, and plasma HIV-1 viral load.

PrEP delivery will be according to the 2016 Kenya PrEP guidelines [46], including measurement of renal function (estimated creatinine clearance >50 mL/min to start PrEP and periodic monitoring over time, aligned to the visit schedule of the study), standard clinical assessment to avoid initiating PrEP during acute HIV-1 infection, and adherence counseling. Subjects will then be randomized at enrollment (Month 0) in a 2:1 fashion to alternating HIV-1 testing at home (Months 3, 9) with in clinic testing (Months 6, 12), translating to clinic visits every 6 months (the self-testing arm), or in-clinic follow-up visits with HIV-1 testing every 3 months (the standard of care arm). Those randomized to self-testing will be further randomly assigned to half receive oral-fluid tests and half blood-based tests (and will use the same type of test throughout their follow-up). Participants in the self-testing arms will get 6 months of PrEP medication at each visit while only those on the clinic based arm will get 3 months of PrEP,

corresponding to enough to last until the next clinic-based visit. Participants in both arms will be counseled on PrEP discontinuation according to Kenya national guidelines (e.g., for HIV-1 uninfected members of HIV-1 serodiscordant couples, 6 months after their partner has begun taking ART, if there are no other HIV-1 risks). Tracing for retention at Months 6 and 12 will be done, particularly to establish PrEP continuation and HIV-1 status. All HIV-1 serodiscordant couples will receive counseling on PrEP as a bridge to ART; infected partners will be encouraged to start ART if not already started **(Tables 2-4).**

As we did in our pilot evaluation (detailed above) those assigned to self-testing will receive training – blood or oral depending on their assignment and will complete one test in clinic on the day of randomization including interpretation of the test result under the guidance of the study staff to increase comfort with the process. Those assigned to HIV-1 self-testing will be provided with two self-testing kits to conduct quarterly testing until their next scheduled visit (i.e., one test kit for the quarterly visit and an extra kit to be used as back-up), and participants will be counseled to use the self-testing kit at a place and time where they will feel comfortable performing the testing (e.g., at home). Participants will also be asked to bring back the used HIV-1 self-test kits to confirm HIV-1 self-testing (though this method has limitations, it will be a proxy measure). As we did for our pilot study, a pictorial information brochure translated into local languages will be provided and a toll-free 24-hour helpline will be provided to call in case of challenges in performing the self-testing or in the event of a positive test result. Participants will be informed that any positive self-test result will need to be confirmed by study staff in accordance with the Kenyan testing algorithm and additional testing as needed (we have used both laboratory-based HIV-1 antigen/antibody enzyme-linked immunoassay testing and HIV-1 RNA PCR to confirm HIV-1 seroconversions in our prior studies and would do so in this project as well, to provide a complete ascertainment of HIV-1 status).

At the final visit (Month 12, for most participants) all participants will be trained on a HIV-1 self-testing method that they had not used and offered an opportunity to self test using the new method, specifically those on the standard arm will be trained on both oral HIV self-testing and blood based HIV self-testing while those on the oral HIV-1 self-testing arm will be trained on the blood based HIV self-testing and those on the blood based HIV-1 self-testing will be trained on the oral HIV-1 self-testing. A HIV-1 testing preference questionnaire will then be administered to assses participants preferences between the three HIV-1 testing methods.

At each study visit, we will offer counseling for participants for HIV-1 testing (pre- and post-testing), HIV-1 infection risk reduction best practices, condom promotion and provision, adherence to HIV-1 medication, adherence to HIV-1 self-testing, and PrEP and ART as HIV-1 prevention strategies.

Table 2. Procedures for HIV-1 uninfected participants: standard of care testing arm

[ ] as

Procedure					
	E)	M3	M6	M9	M12/final visit
Obtain informed consent	X				
Apply inclusion/exclusion criteria, including behavioral and lab eligibility	X				
Collect/update locator information	X	X	X	X	X
Collect demographic information	X				
Collect sexual behavioral information	X		X		X
Collect alcohol and substance use data	X		X		X
Collect depression indicators	X		X		X
Collect HIV-1 risk perception data	X		X		X
Collect general self-efficacy indicators	X		X		X
Provide HIV-1 rapid test results	X	X	X	X	X
Medical history / symptoms information	X	X	X	X	X
Perform physical exam	X	[X]	[X]	[X]	[X]
STI syndromic assessment and management	X	[X]	[X]	[X]	[X]
Offer and provide PrEP sufficient until next visit, instructions; discontinue PrEP 6 months after HIV-1 infected partner initiates ART	X	X	X	X	X
Randomization	X				
Risk reduction counseling and condom promotion & provision	X	X	X	X	X
Contraception counseling and provision/referral	X	X	X	X	X
Adherence counseling	X	X	X	X	X
Provide HIV-1 pre and post-test counseling	X	X	X	X	X
Collect PrEP adherence data	X		X		X
HIV-1 self-testing preferences	X		X		X
Collect antiretroviral-based prevention preference data, information on fertility intentions, other sociobehavioral data to inform PrEP and ART preferences and use	X		X		X
Collect blood specimen for HIV-1 testing and other lab tests as defined here	X	X	X	X	X
Measure serum creatinine			X		X
Dried blood spot collection for PrEP adherence	X		X		X
Measure blood hemoglobin to calibrate measures of PrEP adherence	X		X		X
Plasma for PrEP adherence.	X		X		X
HIV-1 serology (rapid test and, if positive, confirmatory testing according to Kenya policies)	X	X	X	X	X
Urine pregnancy test (women only, as clinically indicated or requested by the participant)	[X]	[X]	[X]	[X]	[X]

indicated

Table 3. Procedures for HIV-1 uninfected participants: HIV-1 self-testing arms

Procedure	E	M6	M12/final visit
Obtain informed consent	X		
Apply inclusion/exclusion criteria, including behavioral and lab eligibility	X		
Collect/update locator information	X	X	X
Collect demographic information	X		
Collect sexual behavioral information	X	X	X
Collect alcohol and substance use data	X	X	X
Collect depression indicators	X	X	X
Collect HIV-1 risk perception data	X	X	X
Collect general self-efficacy indicators	X	X	X
Provide HIV-1 rapid test results	X	X	X
Medical history / symptoms information	X	X	X
Perform physical exam	X	[X]	[X]
STI syndromic assessment and management	X	[X]	[X]
Collect used HIV-1 self test kits		X	X
Offer and provide 6 months of PrEP, instructions; discontinue PrEP 6 months after HIV-1 infected partner initiates ART	X	X	X
Randomization (half will be randomized to blood-based test while the other half will receive oral-based test)	X		
Risk reduction counseling and condom promotion & provision	X	X	X
Contraception counseling and provision/referral	X	X	X
Adherence counseling (at M1, by phone, an adherence check-in)	X	X	X
Provide HIV-1 pre and post-test counseling	X	X	X
Collect PrEP adherence data	X	X	X
Collect HIV-1 self-testing data	X	X	X
HIV self-testing preferences		X	X
Collect antiretroviral-based prevention preference data, information on fertility intentions, other sociobehavioral data to inform PrEP and ART preferences and use	X	X	X
Collect blood specimen for HIV-1 testing and other lab tests as defined here	X	X	X
Measure serum creatinine		X	X
Dried blood spot collection for PrEP adherence	X	X	X
Measure blood hemoglobin to calibrate measure of PrEP adherence	X	X	X
Plasma for PrEP adherence.	X	X	X
HIV-1 serology (rapid test and, if positive, confirmatory testing according to Kenya policies)	X	X	X
Urine pregnancy test (women only, as clinically indicated or requested by the participant)	[X]	[X]	[X]

[ ] as indicated

Table 4. Procedures for index (HIV-1 seropositive) participants at the SINGLE study visit

**Baseline Procedures**
Obtain informed consent
Apply inclusion/exclusion criteria
Collect demographic information
Collect sexual behavior information
Medical history
Risk reduction counseling and condom promotion & provision
Collect ART adherence data
Adherence counseling (if on ART)
Provide HIV-1 pre and post-test counseling
Collect antiretroviral-based prevention preference data, information on fertility intentions, other sociobehavioral data to inform PrEP and ART preferences and use
**Laboratory procedures**
Conduct standard HIV-1 testing
CD4 count
Plasma HIV-1 viral load

Table 5. Procedures for Qualitative data collection (from both study arms).

**Procedure**	**Baseline**	**M6**	**M12/final visit/study end**
In-depth interviews	X	X	X
Focus Group Discussions			X
Health provider interview			X

Seroconversion

Seroconversion will be determined by local HIV-1 testing guidelines. For initially HIV-1 uninfected participants who seroconvert, a plasma sample or dried blood spot will be collected and archived for tenofovir levels and resistance testing. Couples and young women in which the initially HIV-1 uninfected participant seroconverts will be exited from the study but will continue with their normal HIV clinic care follow up as usual. A suspected seroconversion will be confirmed per the Kenyan National Testing Algorithm. In addition, suspected seroconverters will have a blood sample drawn that will be sent to laboratory identified by NASCOP for drug resistance testing as per Kenyan National Guidelines. Laboratory testing for suspected seroconverters is done as per standard clinical care and is not considered part of the study procedures.

Participant retention and withdrawal

Thika site will develop retention methods tailored to and most efficient for the local study setting. Retention activities may include explanation of the study visit schedule and procedural requirements during the informed consent process and re-emphasis at each study visit, collection and updating of locator information, and use of appropriate and timely visit reminder mechanisms (including phone calls and text messages). To provide complete information at the end of the study, efforts will be made to have a final follow-up visit for each participant.

Participants may voluntarily withdraw from the study for any reason at any time. The site Investigator also may withdraw participants from the study in order to protect their safety and/or if they are unwilling or unable to comply with required study procedures. Reasons for withdrawal will be recorded.

Adherence

High adherence is important for PrEP effectiveness in preventing HIV-1 acquisition. Study staff also will provide brief adherence counseling at each scheduled visit, in accordance with Kenya PrEP counseling guidelines.

Data on adherence to the product use regimen will be collected via standardized interviewer-administered questions to ascertain product use. Finally, adherence will also be assessed through batched drug levels at enrollment and study visits at Months 6 and 12. Dried blood spots and plasma from batched drug levels will be shipped to the University of Washington for processing and analysis for tenofovir drug levels. Dried blood spot data are improved when analyzed with standardization against hemoglobin concentrations.

Discontinuation of PrEP

PrEP continuation will be according to Kenya PrEP guidelines. Use of PrEP may be interrupted by the site Investigator due to safety concerns for the participant, use of concomitant medications that could interfere with PrEP or present a safety concern, or if the participant is unable or unwilling to comply with study procedures. All treatment interruptions will be documented.

SAFETY

Multinational studies including the Thika site conducted Partners PrEP Study and Partners Demonstration Project demonstrated that PrEP (including FTC/TDF) was safe for use in heterosexual men and women from Kenya and Uganda. There were no statistically significant differences in the frequency of deaths, serious adverse events, adverse events overall, or key laboratory adverse events (specifically, creatinine elevation and phosphorus decrease) for those receiving PrEP compared to those receiving placebo in the Partners PrEP study.

For the purposes of this study, only serious adverse events (SAEs), for both index (HIV-1 infected) and partner (HIV-1 uninfected) participants, and adverse events felt related to PrEP or self-testing will be documented. SAEs felt to be related to PrEP will result in temporary hold of PrEP. In the case of temporary holds, the hold will continue until the event is stabilized or resolved. If the event resolves, PrEP may be reinitiated at the discretion of the Investigator, resuming safety monitoring. The severity of clinical symptoms will be scored using the DAIDS Table (July 2017 Version) for Grading the Severity of Adult and Pediatric AEs. Reporting on adverse events to relevant IRBs will be according to relevant regulations.

Pregnancy among partner (HIV-1 uninfected) participants

Animal and human data, including from the Partners PrEP Study and Partners Demonstration Project, suggest safety of FTC/TDF when used by HIV-1 infected women during pregnancy and breastfeeding. Other studies are exploring detailed safety of PrEP use in pregnancy. For this study, PrEP will not be discontinued when pregnancy is detected.

HIV-testing safety

HIV-1 testing is an essential component of PrEP delivery to protect patient safety – specifically, to prevent the initiation of PrEP among persons who have already acquired HIV-1 and to prevent continuation of PrEP if HIV-1 is acquired while receiving PrEP. In clinical trials and delivery projects of PrEP, the greatest risk of HIV-1 infection is at the time of initiation – either because of unrecognized chronic HIV-1 infection prior to HIV-1 testing as part of PrEP start or recent (acute) HIV-1 infection acquired just prior to PrEP initiation. Global and national guidelines for PrEP use do not have a consistent standard for the type of test to be used; Kenya, for example, recommends testing according to the national HIV-1 testing algorithm, which uses third-generation HIV-1 antibody based tests, done in sequence (i.e., if a positive first test, then perform a second test for confirmation, and a third as tie-breaker). Antigen-based tests (and HIV-1 RNA testing) are not in widespread use, although antigen-based options are becoming more available.

For the self-testing arms, there is an inherent difference in sensitivity between the oral fluid- and blood-based tests, as detailed above; we recognize this as a potential risk for undetected infection, and ascertainment of the magnitude of that risk will be part of this evaluation. Incident HIV-1 infections are rare among persons offered PrEP (<0.5% per year in our prior work, and essentially 0% among those taking PrEP) and thus HIV-1 acquisition is expected to be very uncommon in this study.

Social harm considerations for HIV-1 self-testing and PrEP

We have extensively considered the risk of social harm related to both PrEP use and HIV-1 self-testing at home, including risks of depression/anxiety and disclosure and stigma. Our extensive experience with longitudinal follow-up of heterosexual HIV-1 serodiscordant couples and women at risk mitigates some of this risk, and we found very little risk of social harms or anxiety related to HIV-1 self-testing in our pilot evaluation, among couples (detailed above). Low evidence of social harms has been reported in other HIV-1 self-testing studies [47]. The 24-hour helpline that that Thika clinic has, and which we used for our pilot study, will be available in case of anxiety, social harm, depression, or a positive test. Analyses of social harm related to self-testing will be done overall, by sex and by relationship status, given the potential for differential gendered and relationship risks (detailed more in Aim 2). In the event of a clinical need (e.g., side effects, symptoms of a sexually transmitted infection), participants will be requested to return to the clinic for care.

DATA AND ANALYSIS

The primary goal of this project is to address key access and cost of delivery challenges for PrEP by using the new modality of HIV-1 self-testing.

Data collection

We will use structured interviews on HIV-1 testing practices and self-reported PrEP adherence (e.g., frequency, ability, self-rating, missed doses). We will use CommCare, an electronic data capture platform, to collect data at the Thika site.

Qualitative data collection

Qualitative data collection will include serial in-depth interviews using pre-piloted semi-structured guides (n=20 men in couples, n=20 women in couples, and n=20 women at risk, from both self-testing modalities and the standard of care randomization arm) and focus group discussions (n=8, stratified by gender and whether or not in a serodiscordant partnership), conducted by experienced social scientists acting in the roles of facilitator and note taker.The semi-structured qualitative interview guide will provide a general structure for discussion but require participants to share their own barriers and facilitators [48]. The serial in-depth interviews will be conducted soon after enrollment to get information on early experiences, at Month 6, and after Month 12 after participants have tested for a couple of visits. The focus group discussions will be conducted at study exit. Interviews will be conducted using the participants preferred language and by consensus in the focus group discussions. The sampling for the qualitative interviews to include a range of possible perspectives (stratified-purposive sampling), to allow for stratification by study arm, gender, HIV-1 serodiscordant relationship status, and other relevant factors that may emerge during the work.

Qualitative discussions will be recorded, transcribed, and translated into English by the study team. Audio recordings will be destroyed by the qualitative team after being transcribed not later than August 2023. Qualitative work will extend our prior work among couples and women at risk, and we will adapt existing questionnaires and topic guides to focus on questions related to HIV-1 testing in the context of PrEP delivery, specifically to gain a deeper understanding of participants' experiences with HIV-1 self-testing, including challenges, benefits, concerns, risks, preferences (self- versus provider-based testing) and intention to use in the future, if available.

Health provider barriers and facilitators to HIV-1 self-testing

Health providers are key to PrEP delivery [49]. Therefore, in addition to self-testing users, we will study operational delivery at the level of providers. We will conduct key informant interviews (n=6-8) with providers at the research clinic (counselors, nurses, clinicians) to understand acceptance, barriers, facilitators, and confidence regarding HIV-1 self-testing in the context of PrEP. Interviews will be conducted at the end of the study. We will document suggestions on how to optimize PrEP delivery through the use of HIV-1 self-testing. We will use similar interview and analysis methods detailed above.

Outcomes

Trial outcomes will be PrEP adherence (PrEP quantity in dried blood spots and PrEP refills), HIV-1 testing completion, and safety (including accuracy of HIV-1 testing, management of side effects, and social harm). **Timing.** All outcomes will be assessed at the Month 6 and 12 clinic visits. The study is powered against a single measurement of adherence (Month 6, see calculations below). **Adherence outcome.** Adherence by PrEP in dried blood spots is defined as the primary trial outcome for the purposes of sample size calculations. Cumulative and recent adherence to PrEP will be measured by concentrations of tenofovir diphosphate (TFV-DP) and emtricitabine triphosphate (FTC-TP), respectively, in a 3 mm punch from a dried blood spot in the laboratory of Dr. Anderson.

TFV-DP and FTC-TP are measured by validated liquid chromatography tandem mass spectrometry (LC-MS/MS), which has become the gold standard for research evaluations of PrEP adherence [10, 11]. High TFV-DP levels will define good adherence. In recent studies, levels ≥1250 fmol/punch (=the median among persons taking 7 doses/week) and ≥700 (=≥4 doses/week, associated with high HIV-1 protection in some studies) have been used. FTC-TP detection with low TFV-DP will distinguish those with only recent (e.g., "white coat dosing") adherence [10, 11, 50]. In addition, we will be standardizing drug levels in dried blood spots by collecting whole blood to measure levels of hemoglobin, which has been validated as a more accurate means of interpretating drug levels.PrEP refills will be measured through data from the clinic's electronic pharmacy system. **Testing and safety outcomes.** HIV-1 testing will be measured as the combination of in-clinic tests and home tests, recorded by self-report and by requesting that completed self-test cartridges be returned to the clinic for validation of self-report. HIV-1 incidence will be measured but is expected to be low (0.2% per year in the Partners Demonstration Project, and 0% for those taking PrEP) and the study would need to be considerably larger to be powered to assess incident HIV-1 (and unnecessary, given the established relationship between PrEP adherence and efficacy); we will assess genotypic HIV-1 resistance among any seroconverters. **PrEP duration.** In our recently-completed Partners Demonstration Project, most couples used PrEP for 9-12 months, justifying our 12 months of follow-up for the couples; those who are established on ART by 6 months will be recommended to discontinue PrEP and their follow-up thereafter for the purposes of PrEP adherence and continuation will be censored. All individuals requiring PrEP for longer than 12 months will continue to receive PrEP through the clinic's services. Analyses for Aim 1, and Aim 2, will be done overall (total population of n=495), separately for serodiscordant couples (n=330) and for women (n=330, half in serodiscordant relationships) – each of these separate subgroups (i.e., couples and women) is well-powered for adherence assessment.

Self-testing to standard of care clinic testing, powered for the PrEP adherence outcome; we hypothesize that HIV-1 self-testing at home will not substantially undermine PrEP use, and thus a non-inferiority design is most appropriate. Most individuals will continue to be eligible for PrEP for 12 months (minus those with a clinical hold, expected to be rare, and those in the couples who for whom the HIV-1 infected partner immediately began ART at baseline and has continued with high adherence); we have powered the study for a single adherence assessment (e.g., Month 6) and increased power will result from repeated measurement of most participants at Month 12. **Primary comparison.** The primary comparison will be self-testing versus clinic testing; the two self-testing modalities will be analyzed together (versus clinic testing) because we hypothesize that the effect on adherence and other outcomes relates to the use of self-tests and frequency of follow-up, not the self-test modality; Aim 2 and 3 are designed to explore potential differences in acceptability and cost between self-testing modalities. **Power.** The trial will be powered for the adherence outcome (measured by detection of PrEP in dried blood spots). Based on our prior work in couples, and published work for women at risk, we estimate ~80% among those seeking to initiate PrEP will achieve blood levels consistent with high PrEP adherence [51, 52]. Thus, if PrEP adherence is 80% in both the standard of care and self-testing arms, with 80% power, 10% loss-to-follow-up, a one-sided 95% confidence interval (common for non-inferiority trials), and a 10% non-inferiority margin, a sample size of n=495 is needed (n=330 self-testing, n=165 standard of care). A 10% non-inferiority margin has been chosen as an important reduction in PrEP use that might be tolerated in order to gain programmatic efficiency through HIV-1 self-testing. We have planned for two sub-analyses with sample sizes of n=330: those in HIV-1 serodiscordant relationships (which will include 165 men and 165 women) and women (which will include the 165 in serodiscordant relationships pus an additional 165 women at risk). These primary sub-analyses will have 80% power to rule out a 12% decrease in adherence (i.e., a slightly greater non-inferiority margin). We have considered whether enrolling n=330 women at risk (rather than n=165, alone or in addition to n=330 couples) would be warranted but feel we can gain important information about both of these populations through the hybrid design, with greater efficiency, in time and in costs. However, we recognize that women outside of serodiscordant relationships may face unique adherence challenges and less frequent follow-up could be truly inferior to quarterly follow-up; thus, among the n=165 women at risk, we will also plan a superiority analysis, for which we will have 80% power to detect a decline in adherence from 80% to 66% (14% lower) with self-testing. For the HIV-1 testing outcome, utilization of self-test kits will be compared between arms; assuming 95% of participants test, we have 80% power to detect a difference of 5% in use of consistent use of self-testing kits (90% vs. 95%). Also, if we end up having to over-enroll HIV-1 uninfected women at HIV-1 risk not in disclosed HIV-1 serodiscordant relationships to compensate for difficulties or delays in enrolling HIV-1 uninfected men and women in HIV-1 serodiscordant relationships, we will have more power to detect outcomes in this important sub-group.

Costing. The costs for a PrEP delivery program that uses HIV-1 self-testing with 6-monthly visits compared to quarterly visits with standard testing will be estimated. Activity-based micro-costing will be conducted and compared by study arm for costs incurred (start-up activities, recruitment, service delivery, monitoring costs, and adherence support) and costs averted (personnel, lab monitoring, incident HIV-1 cases, social, and health benefits). Time and motion studies will be conducted by observing clinic visits, and staff time spent on training on use of home HIV-1 self-testing, counseling clinical procedures, cost of the three types of HIV-1 self-testing kits (blood based provider kit, blood based HIV-1 self-testing kit and oral based HIV-1 self-testing kit) and ART and PrEP delivery. Adjusting for time spent on research activities (e.g., informed consent, research questionnaires), the total time required for PrEP delivery will be estimated. Estimates of cost using the activity-based approach will be compared with the top down (dividing the budget by the number of clients) approach to support scalable estimates of cost. Costs incurred by clients for clinic visits (which contribute to the societal perspective) will be estimated with standard questionnaires that we have used previously.

Model. Mathematical models will be used to simulate health outcomes from a combination of study data and the literature, allowing us to consider clinical outcomes beyond the scope of the prospective cohort. We will adapt our existing compartmental dynamic transmission models (programmed in Matlab) to estimate and compare the impact of each of the interventions on HIV-1 incidence [19, 53, 54]. The model explicitly includes patterns of risk behavior and can account for risk compensation. Our simulation model of HIV-1 progression, transmission, and treatment in HIV-1 serodiscordant couples includes the composition of couples (by sex, age, and CD4 counts), aging, use of ART, conception and pregnancies, variations in coital frequency within stable partnerships and partner change rate. To adapt and parameterize the model, we will use data from our prior studies including the Partners Demonstration Project and the Kenya AIDS Indicator Survey [2, 14]. An alternative modeling approach would be adapting our individual-based HIV-1 model to include PrEP [55]. Assumptions will be as published previously, including our publication on micro-costing work within the Partners Demonstration Project [19].

For key delivery informants

Health providers are key to PrEP delivery [49]. Therefore, in addition to self-testing users, we will study operational delivery at the level of providers. We will conduct key informant interviews (n=6-8) with healthcare providers at the research clinic (counselors, nurses, clinicians) to understand acceptance, barriers, facilitators, and confidence regarding HIV-1 self-testing in the context of PrEP. Interviews will be conducted at the end of the study. We will document suggestions on how to optimize PrEP delivery through the use of HIV-1 self-testing. We will use similar interview and analysis methods detailed above.

Quantitative Analysis

Analyses will be intention-to-treat. HIV-1 testing and PrEP detection in blood will be compared between arms using repeated measures analysis of proportions (e.g., generalized estimating equations [GEE]); PrEP continuation, defined as not missing any refill, will be analyzed as a time-to-event outcome using Cox proportional hazards regression. As noted above, for the couples, those in which the HIV-1 uninfected partner completes PrEP use because the infected partner has initiated and sustained ART for >6 months (the Kenya standard for discontinuing PrEP) will be considered to have successfully used PrEP and will be counted as adherent; similarly, if PrEP is discontinued by the treating clinician for safety reasons (but not adherence reasons), follow-up thereafter will be censored, since the subject will not be able to be assessed for adherence to PrEP. Adjusted analyses will be done as needed, controlling for potential confounders based on our prior work assessing correlates of PrEP use: demographics (e.g., gender, age, educational level), sexual behaviors (e.g., condom use, outside partnerships), medical status (e.g., depression), and beliefs (e.g., risk perception, PrEP efficacy). SAS or R will be used.

For quantitative data, descriptive analyses will be done at baseline and over time, and GEE will be used to test associations between facilitators/barriers and testing preferences outcomes. Analyses will be done to test effect modification by gender and, within women, by serodiscordant couple status

Qualitative Analysis

The transcripts will be reviewed separately by two investigators for completeness and initial theme generation. Coding and analysis will be performed with Atlas.ti, using inductive approach informed by grounded theory [56]. We will then review the results of our coding for consistency of text segmentation and code application with continued inter-coder agreement, and inconsistent results will be reviewed by the coders until consensus is reached and then codes will be grouped together into themes through consensus among coders [57, 58]. We have extensive experience with in-depth interviews with purposefully sampled individuals and couples; for couples we have found dyadic interviews to be a powerful tool to explore joint decision-making and power/gender dynamics [49]. Analyses of the qualitative data will assess how both individuals and dyads respond to HIV-1 self-testing in the context of PrEP, with areas of focus such as gender roles, sexual negotiation, trust, and power. In addition, we will probe delivery, including preference of quarterly versus 6-monthly visit schedule, confidence in HIV-1 self-testing, confidence in blood versus oral fluid, and confidence in HIV-1 testing and PrEP more broadly.

HUMAN SUBJECTS CONSIDERATIONS

The protocol, informed consent forms (for cohort participation and for interviews of providers), and patient education and recruitment materials will be reviewed and approved by the institutional review boards at the University of Washington and at KEMRI. All participants will provide written informed consent before participation in the quantitative and qualitative interviews. Participants will be informed the purpose of the study, the procedures to be followed and the risks and benefits of participation. The consents forms will be translated into Kiswahili. Specifically the participants will be informed that this novel study will answer a critical questions on acceptability, performance and barriers of HIV-1 self testing in the context of implementation of PrEP. Participants will also be informed that the counselors may conduct a home visit to provide additional support after a positive test result if required.

Study oversight

This study will be subject to oversight by an independent data monitoring committee that will periodically review data from the study, including study execution, adherence, HIV-1 incidence, HIV-1 drug resistance, and serious adverse events. Review will be in an unblinded fashion, consistent with the open-label, randomized, unblinded nature of the study. The independent data monitoring committee will provide recommendations to the study team as part of six-monthly reviews. Reports from all reviews will be provided for submission to overseeing IRBs/ECs.

Risks

Partner participants may feel pain or discomfort from phlebotomy if selected for a blood sample archive. Participants may become embarrassed, worried, or anxious when answering behavioral or demographic questions. We have trained counselors who are available through the study to help participants deal with any feelings or questions they may have. The study staff will make every effort to protect participant privacy and confidentiality while you are in the study. However it is possible that participants' involvement in the study could become known to others, and that social harms may result (i.e., because participants could become known as participating in a trial involving HIV-1 infected persons). For example, participants could be treated unfairly or discriminated against, or could have problems being accepted by their families and/or communities.

Benefits

Participants will benefit from ongoing access to this prevention package.

This study aims to provide HIV-1 prevention policy makers with information on how to best implement antiretroviral-based HIV-1 prevention. In addition to the provision of this biomedical method, the study site will provide CHCT, and routine adherence counseling. The outcome of the study will be evidence upon which to based policy guidelines for scaling up HIV-1 prevention centers in Kenya and nearby countries with similar HIV-1

prevention needs. The HIV-1 treatment centers that serve as sites for this study will be models upon which future centers can be based. Summary outcomes from this study will be submitted to overseeing regulatory bodies and will be especially important for the development of normative guidance.

Care for persons identified as HIV-1 infected

This study will identify persons who are infected with HIV-1, either as part of the study screening process or during follow-up of enrolled participants. Study staff will provide participants with their HIV-1 test results in the context of post-test counseling. Persons identified as HIV-1 infected will be referred for care.

Treatment for injury

Participants will be asked to inform the clinic staff if they feel they have been injured because of taking part in the study. Injuries may also be identified during laboratory testing, medical histories, and physical examinations. Treatment for adverse events related to study participation will be provided by the treatment clinic. If treatment is required that is beyond the capacity of the clinic, the clinic staff will refer the participant to appropriate services or organizations that can provide care for the injury.

Study records

Implementation investigators will maintain, and store in a secure manner, complete, accurate, and current study records throughout the study. Study records include administrative documentation and regulatory documentation as well as documentation related to each participant enrolled in the cohort, including informed consent forms, data forms, notations of all contacts with the participant, and all other source documents.

Biometric Identification

Thika site will use a biometric system (fingerprint scanner) to identify participants during follow-up visits. Participants will be offered an informed consent form with the option to accept or decline to have their fingerprint taken. Participants who decline to have their finger print taken will not be excluded from taking part in the study. The finger print database will be destroyed after completion of active follow-up in the study.

Confidentiality

Every effort will be made to protect participant privacy and confidentiality to the extent possible. Personal identifying information will be retained at the local study site

Dissemination Plan

The study team for this award is committed to public dissemination of results of clinical trial, to trial participants, local stakeholders in Kenya, the global scientific community, and US, Kenyan, and global policymakers. Dissemination of study results will follow principles of good participatory practice. The clinical trial will be registered with Clinicaltrials.gov prior to initiation and results will be updated there in a timely fashion. Results will be published in conference abstracts and peer-reviewed journals. Study results will be disseminated through presentations to local stakeholders and policymakers in Kenya, including the Ministry of Health.

REFERENCES

1. UNAIDS. **The Gap Report**. In. Geneva, Switzerland: UNAIDS; 2014.
2. Cherutich P, Kaiser R, Galbraith J, Williamson J, Shiraishi RW, Ngare C, et al. **Lack of knowledge of HIV status a major barrier to HIV prevention, care and treatment efforts in Kenya: results from a nationally representative study**. *PLoS One* 2012; 7(5):e36797.
3. Grant RM, Lama JR, Anderson PL, McMahan V, Liu AY, Vargas L, et al. **Preexposure chemoprophylaxis for HIV prevention in men who have sex with men**. *N Engl J Med* 2010; 363(27):2587-2599.
4. Baeten JM, Donnell D, Ndase P, Mugo NR, Campbell JD, Wangisi J, et al. **Antiretroviral prophylaxis for HIV prevention in heterosexual men and women**. *N Engl J Med* 2012; 367(5):399-410.
5. Thigpen MC, Kebaabetswe PM, Paxton LA, Smith DK, Rose CE, Segolodi TM, et al. **Antiretroviral preexposure prophylaxis for heterosexual HIV transmission in Botswana**. *N Engl J Med* 2012; 367(5):423-434.
6. Choopanya K, Martin M, Suntharasamai P, Sangkum U, Mock PA, Leethochawalit M, et al. **Antiretroviral prophylaxis for HIV infection in injecting drug users in Bangkok, Thailand (the Bangkok Tenofovir Study): a randomised, double-blind, placebo-controlled phase 3 trial**. *Lancet* 2013; 381(9883):2083-2090.
7. U.S. Food and Drug Administration. **FDA approves first drug for reducing the risk of sexually acquired HIV infection**. In; 2012.
8. World Health Organization. **Guideline on when to start antiretroviral therapy and on pre-exposure prophylaxis for HIV**. In. Geneva: WHO; 2015. pp. 78.
9. Baeten JM, Haberer JE, Liu AY, Sista N. **Preexposure prophylaxis for HIV prevention: where have we been and where are we going?** *J Acquir Immune Defic Syndr* 2013; 63 Suppl 2(2):S122-129.
10. Grant RM, Anderson PL, McMahan V, Liu A, Amico KR, Mehrotra M, et al. **Uptake of pre-exposure prophylaxis, sexual practices, and HIV incidence in men and transgender women who have sex with men: a cohort study**. *Lancet Infect Dis* 2014; 14(9):820-829.
11. Anderson PL, Glidden DV, Liu A, Buchbinder S, Lama JR, Guanira JV, et al. **Emtricitabine-tenofovir concentrations and pre-exposure prophylaxis efficacy in men who have sex with men**. *Sci Transl Med* 2012; 4(151):151ra125.
12. McCormack S, Dunn DT, Desai M, Dolling DI, Gafos M, Gilson R, et al. **Pre-exposure prophylaxis to prevent the acquisition of HIV-1 infection (PROUD): effectiveness results from the pilot phase of a pragmatic open-label randomised trial**. *Lancet* 2015; 9(15):00056-00052.
13. Liu AY, Cohen SE, Vittinghoff E, Anderson PL, Doblecki-Lewis S, Bacon O, et al. **Preexposure prophylaxis for HIV infection integrated with municipal- and community-based sexual health services**. *JAMA Intern Med* 2016; 176(1):75-84.
14. Baeten JM, Heffron R, Kidoguchi L, Mugo NR, Katabira E, Bukusi EA, et al. **Integrated delivery of antiretroviral treatment and pre-exposure prophylaxis to HIV-1-serodiscordant couples: A prospective implementation study in Kenya and Uganda**. *PLoS Med* 2016; 13(8):e1002099. doi: 1002010.1001371/journal.pmed.1002099.
15. Gomez GB, Borquez A, Case KK, Wheelock A, Vassall A, Hankins C. **The cost and impact of scaling up pre-exposure prophylaxis for HIV prevention: a systematic review of cost-effectiveness modelling studies**. *PLoS Med* 2013; 10(3):e1001401.
16. Mugo NR, Ngure K, Kiragu M, Irungu E, Kilonzo N. **The preexposure prophylaxis revolution; from clinical trials to programmatic implementation**. *Curr Opin HIV AIDS* 2016; 11(1):80-86.
17. Cambiano V, Miners A, Phillips A. **What do we know about the cost-effectiveness of HIV preexposure prophylaxis, and is it affordable?** *Curr Opin HIV AIDS* 2016; 11(1):56-66.
18. Walensky RP, Park JE, Wood R, Freedberg KA, Scott CA, Bekker LG, et al. **The cost-effectiveness of pre-exposure prophylaxis for HIV infection in South African women**. *Clin Infect Dis* 2012; 54(10):1504-1513.
19. Ying R, Sharma M, Heffron R, Celum CL, Baeten JM, Katabira E, et al. **Cost-effectiveness of pre-exposure prophylaxis targeted to high-risk serodiscordant couples as a bridge to sustained ART use in Kampala, Uganda**. *J Int AIDS Soc* 2015; 18(4 Suppl 3):20013. doi: 20010.27448/IAS.20018.20014.20013. eCollection 22015.
20. Centers for Disease Control and Prevention. **Preexposure prophylaxis for the prevention of HIV infection in the United States - 2014: A clinical practice guideline**. In: US Public Health Service; 2014.
21. Lehman DA, Baeten JM, McCoy CO, Weis JF, Peterson D, Mbara G, et al. **Risk of drug resistance among persons acquiring HIV within a randomized clinical trial of single- or dual-agent preexposure prophylaxis**. *J Infect Dis* 2015; 211(8):1211-1218. doi: 1210.1093/infdis/jiu1677.
22. Parikh UM, Mellors JW. **Should we fear resistance from tenofovir/emtricitabine preexposure prophylaxis?** *Curr Opin HIV AIDS* 2016; 11(1):49-55.

23. U.S. Food and Drug Administration. **First rapid home-use HIV kit approved**. In; 2012.
24. Choko AT, Desmond N, Webb EL, Chavula K, Napierala-Mavedzenge S, Gaydos CA, et al. **The uptake and accuracy of oral kits for HIV self-testing in high HIV prevalence setting: a cross-sectional feasibility study in Blantyre, Malawi**. *PLoS Med* 2011; 8(10):e1001102.
25. Choko AT, MacPherson P, Webb EL, Willey BA, Feasy H, Sambakunsi R, et al. **Uptake, accuracy, safety, and linkage into care over two years of promoting annual self-testing for HIV in Blantyre, Malawi: a community-based prospective study**. *PLoS Med* 2015; 12(9):e1001873.
26. UNITAID. **Increase in number of countries adopting HIV self-testing policies**. In; 2016.
27. Johnson CC, Kennedy C, Fonner V, Siegfried N, Figueroa C, Dalal S, et al. **Examing the effects of HIV self-testing compared to standard HIV testing services: a systematic review and meta-analysis**. *J Int AIDS Soc* 2017; 20:21594.
28. Kenya Ministry of Health. **Kenya HIV prevention revolution road map: Count down to 2030**. In; 2014.
29. Bunnell R, Mermin J, De Cock KM. **HIV prevention for a threatened continent: implementing positive prevention in Africa**. *JAMA* 2006; 296(7):855-858.
30. Marum E, Taegtmeyer M, Chebet K. **Scale-up of voluntary HIV counseling and testing in Kenya**. *JAMA* 2006; 296(7):859-862.
31. Geldsetzer P, Ortblad K, Barnighausen T. **The efficiency of chronic disease care in sub-Saharan Africa**. *BMC Med* 2016; 14(1):127.
32. Baeten JM, Donnell D, Mugo NR, Ndase P, Thomas KK, Campbell JD, et al. **Single-agent tenofovir versus combination emtricitabine plus tenofovir for pre-exposure prophylaxis for HIV-1 acquisition: an update of data from a randomised, double-blind, phase 3 trial**. *Lancet Infect Dis* 2014; 14(11):1055-1064.
33. Murnane PM, Celum C, Mugo N, Campbell JD, Donnell D, Bukusi E, et al. **Efficacy of preexposure prophylaxis for HIV-1 prevention among high-risk heterosexuals: Subgroup analyses from a randomized trial**. *AIDS* 2013; 27(13):2155-2160.
34. Heffron R, Mugo N, Were E, Kiarie J, Bukusi EA, Mujugira A, et al. **Preexposure prophylaxis is efficacious for HIV-1 prevention among women using depot medroxyprogesterone acetate for contraception**. *AIDS* 2014; 28(18):2771-2776. doi: 2710.1097/QAD.0000000000000493.
35. Murnane PM, Heffron R, Ronald A, Bukusi EA, Donnell D, Mugo NR, et al. **Pre-exposure prophylaxis for HIV-1 prevention does not diminish the pregnancy prevention effectiveness of hormonal contraception**. *AIDS* 2014; 28(12):1825-1830. doi: 1810.1097/QAD.0000000000000290.
36. Mugo NR, Hong T, Celum C, Donnell D, Bukusi EA, John-Stewart G, et al. **Pregnancy incidence and outcomes among women receiving preexposure prophylaxis for HIV prevention: a randomized clinical trial**. *JAMA* 2014; 312(4):362-371. doi: 310.1001/jama.2014.8735.
37. Mugwanya KK, Wyatt C, Celum C, Donnell D, Mugo NR, Tappero J, et al. **Changes in glomerular kidney function among HIV-1-uninfected men and women receiving emtricitabine-tenofovir disoproxil fumarate preexposure prophylaxis: a randomized clinical trial**. *JAMA Intern Med* 2015; 175(2):246-254. doi: 210.1001/jamainternmed.2014.6786.
38. Mugwanya K, Baeten J, Celum C, Donnell D, Nickolas T, Mugo N, et al. **Low risk of proximal tubular dysfunction associated with emtricitabine-tenofovir disoproxil fumarate preexposure prophylaxis in men and women**. *J Infect Dis* 2016; 214(7):1050-1057.
39. Mugwanya KK, Wyatt C, Celum C, Donnell D, Kiarie J, Ronald A, et al. **Reversibility of glomerular renal function decline in HIV-uninfected men and women discontinuing emtricitabine-tenofovir disoproxil fumarate pre-exposure prophylaxis**. *J Acquir Immune Defic Syndr* 2016; 71(4):374-380.
40. Karim SS, Karim QA. **Antiretroviral prophylaxis: a defining moment in HIV control**. *Lancet* 2011; 378(9809):e23-25.
41. Cohen J. **AIDS research. Complexity surrounds HIV prevention advances**. *Science* 2011; 333(6041):393.
42. Ngure K, Heffron R, Mugo N, Thomson KA, Irungu E, Njuguna N, et al. **Feasibility and acceptability of HIV self-testing among pre-exposure prophylaxis users in Kenya**. *J Int AIDS Soc* 2017; 20(1):1-8.
43. Masters SH, Agot K, Obonyo B, Napierala Mavedzenge S, Maman S, Thirumurthy H. **Promoting partner testing and couples testing through secondary distribution of HIV self-tests: a randomized clinical trial**. *PLoS Med* 2016; 13(11):e1002166.
44. Mugo PM, Micheni M, Shangala J, Hussein MH, Graham SM, Rinke de Wit TF, et al. **Uptake and acceptability of oral HIV self-testing among community pharmacy clients in Kenya: a feasibility study**. *PLoS One* 2017; 12(1):e0170868.
45. Unitaid. **Market and technology landscaep: HIV rapid diagnostic tests for self-testing**. In. Edited by Services JE. 4th edition ed. Geneva: Unitaid; 2018.

46. National AIDS & STI Control Programme. **Guidelines on the use of antiretroviral drugs for treating and preventing HIV infection in Kenya**. In: Ministry of Health, Kenya; 2016.

47. Brown AN, Djimeu EW, Cameron DB. **A review of the evidence of harm from self-tests**. *AIDS Behav* 2014; 18 Suppl 4(4):S445-449.

48. Office of Behavioral and Social Science Research. **Qualitative methods in health research: opportunities and considerations in application and review.** . In. Bethesda, MD: National Institutes of Health; 2001.

49. Ngure K, Heffron R, Curran K, Vusha S, Ngutu M, Mugo N, et al. **I knew I would be safer. Experiences of Kenyan HIV serodiscordant couples soon after pre-exposure prophylaxis (PrEP) initiation**. *AIDS Patient Care STDS* 2016; 30(2):78-83.

50. Castillo-Mancilla J, Seifert S, Campbell K, Coleman S, McAllister K, Zheng JH, et al. **Emtricitabine-triphosphate in dried blood spots as a marker of recent dosing**. *Antimicrob Agents Chemother* 2016; 60(11):6692-6697.

51. Bekker L-G, Hughes J, Amico R, Roux S, Hendrix C, Anderson PL, et al. **HPTN 067/ADAPT Cape Town: a comparison of daily and nondaily PrEP dosing in African women**. In: *CROI 2015: Conference on Retroviruses and Opportunistic Infections*. Seattle, USA 2015. pp. Abstract 978LB.

52. Baeten JM, Heffron R, Kidoguchi L, Mugo NR, Katabira E, Bukusi EA, et al. **Integrated delivery of antiretroviral treatment and pre-exposure prophylaxis to HIV-1-serodiscordant couples: a prospective implementation study in Kenya and Uganda**. *PLoS Med* 2016; 13(8):e1002099.

53. Ying R, Sharma M, Celum C, Baeten JM, van Rooyen H, Hughes JP, et al. **Home testing and counselling to reduce HIV incidence in a generalised epidemic setting: a mathematical modelling analysis**. *Lancet HIV* 2016; 3(6):e275-282. doi: 210.1016/S2352-3018(1016)30009-30001.

54. Sharma M, Farquhar C, Ying R, Krakowiak D, Kinuthia J, Osoti A, et al. **Modeling the cost-effectiveness of home-based HIV testing and education (HOPE) for pregnant women and their male partners in Nyanza Province, Kenya**. *J Acquir Immune Defic Syndr* 2016; 72 Suppl 2(72):S174-180.

55. Smith JA, Sharma M, Levin C, Baeten JM, van Rooyen H, Celum C, et al. **Cost-effectiveness of community-based strategies to strengthen the continuum of HIV care in rural South Africa: a health economic modelling analysis**. *Lancet HIV* 2015; 2(4):e159-168. doi: 110.1016/S2352-3018(1015)00016-00018.

56. Glaser BC, Strauss AL. **The Discovery of Grounded Theory: Strategies for Qualitative Research**. New York: Aldiric de Gruyter; 1967.

57. Barbour RS. **Checklists for improving rigour in qualitative research: a case of the tail wagging the dog?** *Bmj* 2001; 322(7294):1115-1117.

58. Mays N, Pope C. **Rigour and qualitative research**. *Bmj* 1995; 311(6997):109-112.

HIV self-testing to improve the efficiency of PrEP delivery

JiPime-JiPrEP Study

Statistical Analysis Plan

Confidential

Version 4.3, 28 March 2022

Prepared by:

Katherine K. Thomas, MSs
Katrina Ortblad, ScD, MPH
Ashley Bardon, MPH

International Clinical Research Center
University of Washington

Table of Contents

1. Introduction ... 3
2. Study rationale ... 3
3. Study overview and objectives ... 3
   - 3.1. Study overview ... 3
   - 3.2. Study design ... 4
   - 3.3. Eligibility criteria ... 4
   - 3.4. Study visits ... 4
4. Study endpoints ... 5
   - 4.1. Primary objective outcomes ... 5
     - 4.1.1. Primary ... 5
   - 4.2. Secondary outcomes ... 6
5. Sample size justification ... 7

5.1. Original sample size calculation ... 7
   - 5.2. Sample size re-calculation ... 7
6. Randomization and masking ... 8
   - 6.1. Randomization ... 8
   - 6.2. Masking ... 8
7. Data collection ... 9
   - 7.1. Database ... 9
   - 7.2. Participant identifiers ... 9
8. Statistical considerations ... 9
   - 8.1. Missing data ... 9
   - 8.2. Multiple comparisons ... 10
   - 8.3. Analysis sets ... 10
9. Interim monitoring ... 10
10. Data analysis ... 10
    - 10.1. Overview of data analyses ... 10
    - 10.2. Baseline characteristics ... 10
    - 10.3. Analysis of primary objective outcomes ... 10
    - 10.4. Analysis of secondary efficacy variables ... 11
    - 10.5. Sub-group analyses ... 11
11. Adverse Events ... 12
12. Changes to the SAP after unblinding ... **13**

1. Introduction

This document (Statistical Analysis Plan, SAP) describes the planned analysis and reporting for the **HIV self-testing to improve the efficiency of PrEP delivery (i.e., JiPime-JiPrEP) study**, a joint collaboration between the University of Washington and the Jomo Kenyatta University of Agriculture and Technology (R01MH113572, MPIs: Mugo/Ngure). It includes specifications for the statistical analyses and tables to be prepared for the final study reporting.

The planned analyses described in this SAP will be included in future manuscripts. Note, however, that exploratory analyses not necessarily identified in this SAP may be performed to support the analysis. All post-hoc or unplanned analyses which have not been delineated in this SAP will be clearly documented as such in the final study reporting, manuscripts, or any other document or submission.

2. Study rationale

Pre-exposure prophylaxis (PrEP) and HIV-1 self-testing are new and powerful HIV-1 prevention tools; delivering these strategies will require approaches that are time- and cost-efficient, for patients, care providers, and the health care system. In a highly innovative study bringing these two new tools together, we propose to use HIV-1 self-testing to reduce the frequency of clinic visits for persons taking PrEP, and we will evaluate the effectiveness and safety of our approach using a randomized, non-inferiority trial among women and men initiating PrEP in Kenya. We hypothesize that using HIV-1 self-testing to replace frequent clinic visits for persons on PrEP will not reduce PrEP adherence or continuation of use, will be highly acceptable to patients and providers, and will be associated with reduced health system costs.

3. Study overview and objectives

3.1. Study overview

**Protocol title:**	HIV self-testing to improve the efficiency of PrEP delivery
**Short title:**	JiPime-JiPrEP [test yourself-PrEP yourself]
**Design:**	This study is a randomized non-inferiority trial
**Study arms:**	<u>Intervention:</u> Reduced clinic visits (6-monthly) with HIV-1 test + HIV-1 self-tests (either oral-fluid or blood-based) and a 6-month PrEP supply<br><br><u>Standard-of-care:</u> Standard clinic visits (3-montly) with HIV-1 test + 3-month PrEP supply
**Randomization:**	1:1:1 intervention (blood): intervention (oral fluid): standard-of-care
**Population:**	women and men who recently initiated PrEP (<1 month), in 3 groups:<br>1. HIV-1 uninfected men in a serodiscordant couple<br>2. HIV-1 uninfected women in a serodiscordant couple<br>3. HIV-1 uninfected women at HIV-1 risk, not in a serodiscordant couple
**Sample size:**	495 in total*<br>• 165 HIV-1 uninfected men in a serodiscordant couple<br>• 130 HIV-1 uninfected women in a serodiscordant couple<br>• 200 HIV-1 uninfected women at HIV-1 risk not in a serodiscordant couple<br><br>** The enrollment numbers for HIV-1 uninfected women in and not in a serodiscordant couple were adjusted due to challenges in enrolling HIV-1 uninfected women in a serodiscordant couple and to gain additional information on PrEP use in HIV-1 uninfected women not in a known serodiscordant couple – a priority population for HIV-1 prevention services.*
**Follow-up:**	6 and 12 months (all participants) + 3 and 9 months (standard-of-care only)
**Study site:**	Single PHRD clinic (Thika, Kenya)
**Primary**	We will test the use of HIV-1 self-testing to decrease the frequency and burden of clinic

**objective:**	visits for PrEP while resulting in equivalent adherence and HIV testing.
**Secondary objectives:**	We will test whether the use of HIV-1 self-testing affects recent abuse by a sexual partner, the prevalence of depression, participants' self-efficacy, HIV-1 risk-related sexual behaviors, PrEP disclosure, and HIV testing preferences, compared to standard-of-care PrEP delivery (a 3-month PrEP drug supply).

3.2. Study design

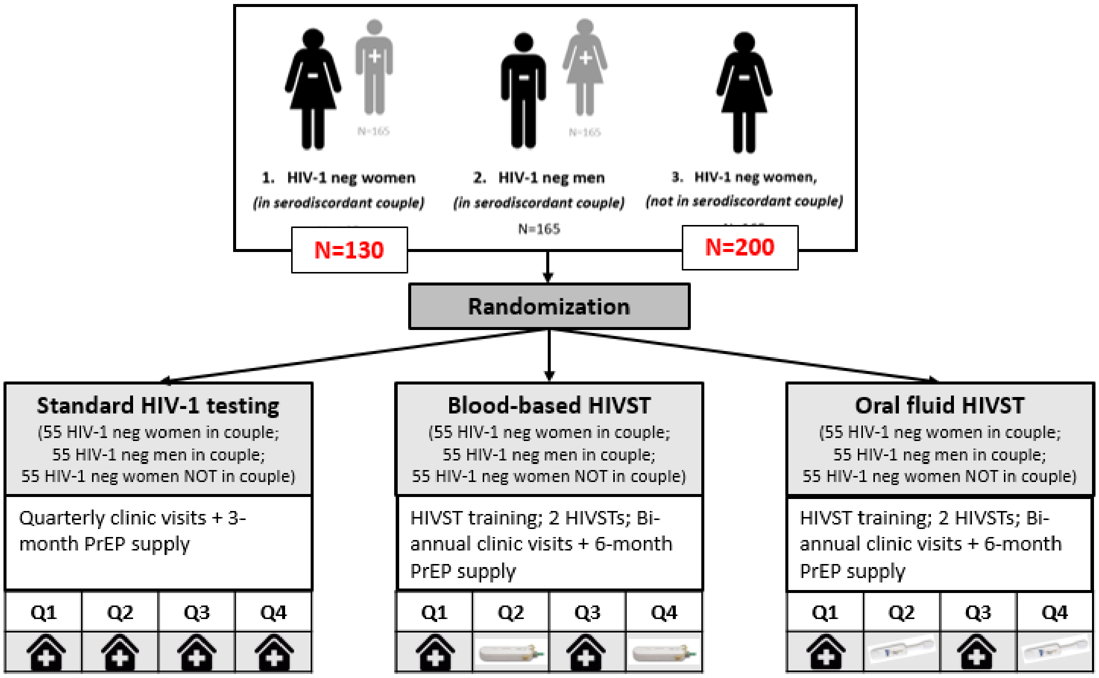

3.3. Eligibility criteria

Inclusion criteria	Exclusion criteria
• Age >= 18 years<br>• HIV-1 uninfected (rapid test)<br>• Not currently enrolled in other trial<br>• Taking PrEP (1 month) and planning to continue<br>• Willing to be randomized to one of the study arms	• Unable to provide written informed consent<br>• Contraindication to use TDF+/-FTC/3TC

3.4. Study visits

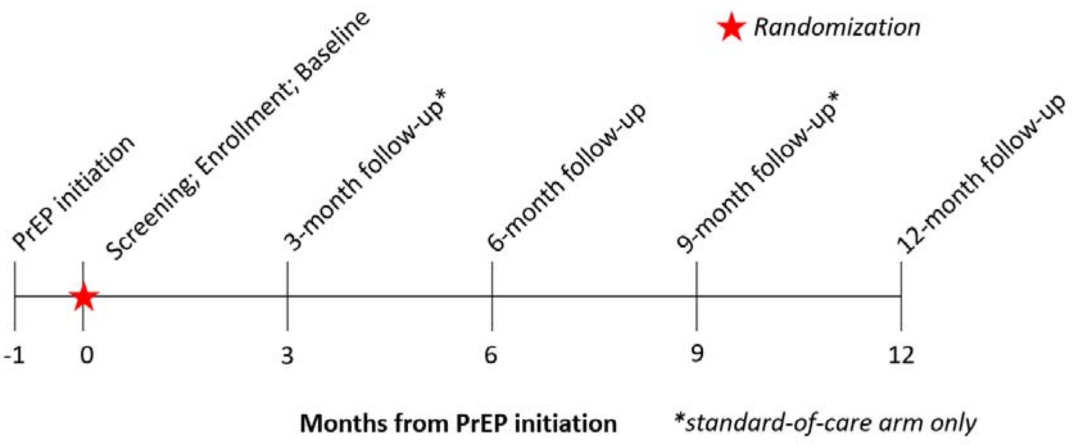

4. Study endpoints

We have registered all primary and secondary trial outcomes on ClinicalTrials.gov (ID: NCT03593629)

4.1. Primary objective outcomes

4.1.1. Primary

The primary objective will be tested using three primary outcomes: 1) HIV-1 testing, 2) persistence in refilling PrEP, and 3) PrEP adherence (all measured at 6 months). We selected the 6-month measurements for our primary outcome measurements because for participants in serodiscordant couples, their sexual partner might have achieved HIV-1 viral suppression by 6 months, as which time there may no longer be a need for the HIV-1 uninfected partner to continue using PrEP.

In order to include all randomized participants when analyzing primary endpoints, we will impute those not contributing a response at the 6 month time point as not achieving the outcome (i.e., we will impute missing data = "fail" for each outcome). This means that those who do not return to study visits (are not "retained" at the study visit) or for other reason do not contribute a response, are counted as not achieving the outcome.

<u>Retention window (6 months)</u>: While most participants return for follow-up close to the scheduled 6-month visit date, operationally the scheduling window for this visit opens 2 weeks prior this scheduled visit date and closes 2 weeks before the next scheduled visit date (at 270 days post enrollment for participants in the SOC arm and at 360 days post enrollment for participants in the HIVST arms). For the purposes of analysis, we will use follow-up visits assigned as 6-month visits by study staff (using the guidelines above) for our primary analysis. Additionally, we will analyze an "on-time retention" window, defined as less than 21 days post the scheduled 6-month visit date. While the "on-time retention" window is less inclusive, it has the benefit of being the same window in both intervention and control groups.

**HIV-1 testing[1]:**	Any self-reported HIV-1 testing (in-clinic tests and home tests, if applicable), between the enrollment and 6-month visit (binary outcome = yes/no, denominator = all randomized subjects, missing = no).
**Persistence in refilling PrEP[1]:**	The proportion of enrolled participants (binary outcome = refilled/not refilled, denominator = all randomized subjects, missing = not refilled) that return to the clinic and refill their PrEP medication, measured using clinic electronic dispensing data.

**PrEP adherence[1]:**	Adherence to PrEP will be measured by concentrations of tenofovir diphosphate (TFV-DP) in a 3 mm punch from a dried blood spot by liquid chromatography tandem mass spectrometry (LC-MS/MS) at the participant's 6-month visit. We will measure PrEP adherence using DBS samples in two ways:<br>• [Primary]: Any detection of TFV-DP (above the limit of quantification) (binary outcome = yes/no, denominator = all randomized subjects, missing = not detected)<br>• [Secondary]: Concentration of TFV-DP $\geq$700 fmol (binary outcome = yes/no, denominator = all randomized subjects, missing = not detected)

[1]We will measure these outcome for both the as-assigned" retention and "on-time" 6-month retention windows (described above) and assume missing = fail.

4.2. Secondary outcomes

We will additionally test whether, compared to SOC PrEP delivery (a 3-month PrEP drug supply), the delivery of a 6-month PrEP drug supply + HIVST affects the primary outcomes (described above) when evaluated at 12 months. We will also measure the effect of the interventions on other secondary outcomes, including: recent abuse by a sexual partner, the prevalence of depression, participants' self-efficacy, HIV risk-related sexual behaviors, PrEP disclosure, and HIV testing preferences at 6 and 12 months.

For the secondary outcomes that are the same as the primary (e.g., HIV-1 testing, persistence in refilling PrEP, and PrEP adherence), we will measure the outcomes in the two different retention windows (described in section 4.1.1), and assume missing = failure. For the secondary outcomes that are unique from the primary outcomes, we will use both 6- and 12-month measurements and will not restrict any analyses to the 'on time' windows and will not impute missing values as failures.

If PrEP is discontinued by the treating clinician for safety reasons (but not adherence reasons), follow-up thereafter will be censored, since the subject will not be able to be assessed for adherence to PrEP.

**HIV-1 testing[1]:**	1. Any self-reported HIV-1 testing (in-clinic tests and home tests, if applicable), past 6 months<br>2. Two or more self-reports of HIV-1 testing between the enrollment and 12-month visit<br>(binary outcomes = yes/no, denominator = all randomized subjects, missing = no).
**Persistence in refilling PrEP[1]:**	1. The proportion of enrolled participants that return to the clinic and refill their PrEP medication, measured using clinic electronic dispensing data, at their 12-month visit.<br>2. The proportion of enrolled participants that return to the clinic and refill their PrEP medication, measured using clinic electronic dispensing data, at both their 6- and 12-month visits.<br>(binary outcomes = refilled/not refilled, denominator = all randomized subjects, missing = not refilled)
**PrEP adherence[1]:**	[See definition in primary outcomes sub-section]
**HIV-1 incidence:**	The proportion of participants (binary outcome) that test HIV-1 positive since trial enrollment (we expect this to be very low due to our small sample size, especially considering that all participants are prescribed PrEP). We will assess prevalence of genotypic HIV-1 drug resistance among any seroconverters.
**Recent abuse, by sexual partner:**	The proportion of participants (binary outcome) that self-report verbal, physical, or emotional abuse by a sexual partner.
**Prevalence of depression:**	The proportion of participants (binary outcome) that report depressive symptoms. Determined using the Patient Health Questionnaire-9 item (PHQ-9) depression scale. A 0-

	27 point scale where scores 10 or greater can be categorized as likely depression.
**Self-efficacy:**	Measured using the General Self-Efficacy Scale (GSE), which is correlated to emotion, optimism, work satisfaction, to measure self-efficacy. The scale ranges from 10-40 points - higher scores indicate more self-efficacy (continuous outcome).
**Number of sexual partners:**	Self-reported number of sexual partners in the past month (numeric outcome).
**Inconsistent condom use:**	Measured by asking participants how many times they had sex in the past month and how many times a condom was used. If condoms were not used every time, condom use was categorized as inconsistent (binary outcome).
**PrEP disclosure:**	The proportion of participants (binary outcome) that report that at least one other person (besides one's main sexual partner in serodiscordant couples) is aware they are taking PrEP.
**HIV-1 testing preferences:**	Participants report their preference for HIV testing from the following options: blood-based HIV-1 self-testing, oral-fluid HIV-1 self-testing, and HIV testing at a standard health care clinic (categorical outcome).

[1]These outcomes (same as the primary, but measured at 12 months) will be measured at 12 months only.

5. Sample size justification

5.1. Original sample size calculation

Primary analyses: The trial will be powered for the primary adherence outcome (measured using any detection of TFV-DP in dried blood spots) at 6 months. Based on our prior work in HIV-1 serodiscordant couples and women at HIV risk, we estimate ~80% among those seeking to initiate PrEP will achieve blood levels consistent with high PrEP adherence. Thus, if PrEP adherence is 80% in both the standard of care and self-testing arms, with 10% loss-to-follow-up, a one-sided 95% confidence interval (common for non-inferiority trials), and a 10% non-inferiority margin, the planned sample size of N=495 (N=330 HIVST arms, N=165 SOC arm) provides 80% power. A 10% non-inferiority margin has been chosen as an important reduction in PrEP use that might be tolerated in order to gain programmatic efficiency through HIV-1 self-testing. Counting those LTFU as nonadherent means all participants randomized contribute.

Sub-group analyses. We planned for three sub-group analyses with: 1) HIV-1 serodiscordant couples (including 165 men and 130 women, 295 participants in total), 2) women (including 130 in serodiscordant couples plus an additional 200 women at risk, 330 women in total), and 3) women outside of serodiscordant couples (including 200 women at risk). The sub-analyses in serodiscordant couples and in all women will have 80% power to rule out a 12% decrease in PrEP adherence (i.e., a slightly greater non-inferiority margin).

We recognize that women outside of serodiscordant couples (N=200) may face unique adherence challenges and less frequent follow-up could be truly inferior to quarterly follow-up; thus, we also plan a superiority analysis for these women. In this analysis, we will have 80% power to detect a decline in PrEP adherence from 80% to 66% (14% lower) with self-testing.

5.2. Sample size re-calculation

Primary analyses: The primary adherence analysis has been redesigned to impute those LTFU as nonadherent, so that all participants randomized will contribute to the analysis. With a 10% non-inferiority margin, if PrEP adherence is 80% in both the standard of care and self-testing arms, our total sample size of N=495 will provide 83.6% power to rule out a greater than 10% decrease in adherence. However, counting those missing as nonadherent will likely lead to <80% considered adherent. If 70% are adherent in each arm, we will have 74% power to rule out

more than a 10% difference. While power <80% is not ideal, the trade-off of including all participants in the analysis is an important consideration in this implementation science study.

Sub-group analyses. We have revised our plan to use the same clinically important non-inferiority margin of 10% for the subgroups. Power is not high when limited to any one subgroup, as is common in a clinical trial powered for analysis in the entire study population: if we assume PrEP adherence is 80% in SOC, to rule out a 10% decrease in PrEP adherence we will have 65% power among HIV-1 serodiscordant couples (N=295), 69% power among all women (N=330) and 51% power among women outside of serodiscordant couples (N=200). If we assume PrEP adherence is 70% in SOC, power will be 55% among HIV-1 serodiscordant couples, 59% among all women. and 43% among women outside of serodiscordant couples. Among the women outside of serodiscordant couples (N=200), we will also have 80% power to detect a decline in PrEP adherence from 80% to 59.8%, or from 70% to 48.4%.

6. Randomization and masking

6.1. Randomization

Randomization details. A list of sequential randomization assignments will be prepared for each study population: 1) HIV-1 uninfected men in HIV-1 serodiscordant couples, 2) HIV-1 uninfected women in HIV-1 serodiscordant couples, and 3) HIV-1 uninfected women at-risk for HIV-1 who are not in disclosed HIV-1 serodiscordant couples. Randomization will occur in a 1:1:1 fashion to: 1) blood-based HIV-1 testing at home (2 test kits distributed at each clinic visit) and in-clinic follow-up visits (including in-clinic testing) every 6 months, 2) oral-fluid HIV-1 testing at home (2 test kits distributed at each clinic visit) and in-clinic follow-up visits (including in-clinic testing) every 6 months, or 3) HIV-1 testing at in-clinic follow-up visits (including in-clinic testing) every 3 months (standard-of-care).

Randomization list. The randomization list was prepared using variable-sized blocks by a UW statistician and is stored in a password-protected electronic file on a UW server. The randomization list consists of a unique identifier for each study population and participant (see Section 7.2), together with the assignment to study arm.

Randomization implementation. Randomization will be done at the enrollment visit, which will occur approximately one month after the participants have begun taking PrEP. At the time of randomization, participants will open on opaque randomization envelope, given to them by a study pharmacist, that has their study arm assignment inside.

6.2. Masking

This study is unmasked. To minimized implementation bias, we have implemented procedures that standardize participant contact across the study arms:

Follow-up period	Standard-of-care arm	HIV self-testing arms
**3-month**	★	--
**6-month**	★	★
**9-month**	★	--
**12-month***	★	★

-- **DO NOT** contact participants because they do not have a scheduled visit

★ Standard clinical procedures for reminding individuals of their appointment: Call one day after missed scheduled visit date then repeat seven days after the first call. An alternative contact, as listed in the locator form, will be called during the second week (after the repeat call made 7 days after the first attempt to contacting participant) if participant fails to answer phone calls from the clinic.

*At 12-months, if participants do not returned for their scheduled clinic visit after standard contact procedures have been implemented and 3 months have passed, we will note this and engage in more intensive efforts to follow-up with this participants (including home visits) so that we can collect end-line data (including a DBS sample) from participants.

7. Data collection

7.1. Database

All quantitative data will be collected electronically in face-to-face interviews with trained Thika HIV-1 counselors, clinicians, and pharmacists. We will use CommCare (Dimagi, Cambridge, USA), an electronic data collection platform, to collect the quantitative data and will upload this data to CommCare's secure server daily. A team of data experts in both Thika and Seattle will monitor the data as it is coming in, and Seattle team will generate weekly data quality reports that will be shared with the Thika team for review and feedback.

7.2. Participant identifiers

Participant identification numbers have the following format: **53-18-XXX-Y-Z**.

**53**	Thika site code
**18**	Protocol number
**XXX**	Sequential digits, specific to participant groups:<br>• **001-300** = HIV-1 negative men in serodiscordant couples<br>• **301-600** = HIV-1 negative women in serodiscordant couples<br>• **601-999** = HIV-1 negative women not in serodiscordant couples
**Y**	Specifies participant group:<br>• **1** = HIV-1 negative men in serodiscordant couples<br>• **2** = HIV-1 negative women in serodiscordant couples<br>• **3** = HIV-1 negative women not in serodiscordant couples<br>• **4** = HIV-1 positive women in serodiscordant couples<br>• **5** = HIV-1 negative man in serodiscordant couple
**Z**	Check digit, a random number: **1-9**

8. Statistical considerations

8.1. Missing data

For all primary outcomes (e.g., HIV-1 testing, persistence in PrEP refilling, and PrEP adherence) we will assume that missing equals failure; for participants not retained in the relevant 6-month (or 12-month) retention windows described in section 4.1.1, the response will be considered missing (and therefore failure). As a potential sensitivity analysis, we may impute missing outcomes using information gleaned through extensive follow-up of participants who miss their 12-month PrEP visit and phone-based surveys conducted during the periods of COVID-19 lockdown, and controlling for potential confounders in our analyses.

8.2. Multiple comparisons

An alpha of 0.05 will be used for the primary analyses and pre-specified secondary analyses. For pre-specified secondary analyses, we will report both the p-value and the number of pre-specified analyses performed.

8.3. Analysis sets

Data sets. Data sets for analysis will be produced by Katrina Ortblad, Dorothy Mangale, and Ashley Bardon. They will be .dta or .csv files containing a single header line whose variable names match those coded in CommCare. All missing values will be coded using "999". Codes for categorical variables (e.g., 0 for "No" and 1 for "Yes") will be used instead of character strings whenever possible.

Data codebook. A detailed codebook will be prepared, containing for each variable the form from which the variable derived, the text of the question, and all possible values for that variable with their coding. All codes and character strings representing categorical factors will be defined in the codebook.

9. Interim monitoring

The study will be monitored by a Data Scientific and Monitoring Board (DSMB) approximately every six months. The project director and statistician will generate both an open and closed report (statistician only) that will be shared with the DSMB prior to the meeting. The DSMB will give recommendations based on the report and accompanying presentation, and all recommendations and meeting minutes will be reported to the UW and Kenyan IRBs.

10. Data analysis

10.1. Overview of data analyses

Analyses. All analyses comparing randomization arms will be by intention-to-treat. The primary comparison will be self-testing versus clinic testing; the two self-testing modalities will be analyzed together (versus SOC in-clinic testing) because we hypothesize that the effect on adherence and other outcomes relates to the use of self-tests and frequency of follow-up, not the self-test modality.

Model adjustments. Models comparing randomized arms will include study arm as the primary predictor in the model, and will adjust only for study population (male in partnership, female in partnership, or female not within partnership). Supplemental, adjusted analyses also will be performed where potential confounders are found to differ at baseline. Potential confounders considered will be based on our prior work assessing correlates of PrEP use: demographics (e.g., gender, age, educational level), sexual behaviors (e.g., condom use, outside partnerships), medical status (e.g., depression), and beliefs (e.g., risk perception, PrEP efficacy). Models containing more than one time point (e.g., 6 month and 12 month data in one analysis) will adjust for time point.

Significance. Significance will be assessed using a two-tailed test at the 0.05 level.

10.2. Baseline characteristics

Baseline characteristics will be described by study arm and study population. These will include demographic variables, HIV-1 testing history, sexual behaviors, and history of intimate partner violence.

10.3. Analysis of primary objective outcomes

To test the study's primary objective we will evaluate non-inferiority of the combined HIVST groups against SOC, for each of the primary objective outcomes: 1) self-reported HIV-1 testing, 1) persistence in refilling PrEP, and 3)

PrEP adherence (any detection of TFV-DP in DBS samples) at 6 months. At 6 months, we will measure these outcomes using two different retention windows: 1) "as-assigned" retention and 2) "on-time" retention. If participants are not retained in care (i.e., the outcome is missing when visit is restricted to the specific retention window), then we will impute the outcome as described in *section 4.1,* describing primary endpoints.

The proportion of participants with each outcome in each of the retention windows will be described by randomized group. Our hypothesis is that the combined HIVST groups will be non-inferior to the SOC group for each of the specified primary objective outcomes. Statistical comparison will be a one-sided non-inferiority comparison, using a binomial regression model with identity link to estimate the risk difference (RD) for the outcome in the HIVST arm compared to SOC. If we encounter problems with model convergence, we will instead use a linear regression model (Gaussian errors and identity link) modified with robust standard errors to allow valid inference in the context of misspecification of the error structure as Gaussian rather than binomial. If the one-sided 95% CI for the RD (HIVST – SOC) excludes values below -10%, then the results will be interpreted as showing that the HIVST groups were non-inferior to SOC. If the 95% CI includes values below -10%, then HIVST is not non-inferior. We will test different retention windows in various sensitivity analyses, including a sensitivity analysis where outcomes are not restricted to different retention windows.

(SOURCE: Naimi AI, Whitcomb BW. Estimating risk ratios and risk differences using regression. *American Journal of Epidemiology*. 2020; 189(6):508-510)

Secondary outcomes consisting of the same outcomes as above, but applied to the 12 month visit, will be conducted using the same methods as the primary outcomes at 6 months. Additionally, we will conduct secondary analyses that compare primary outcomes for individuals in each arm vs. each other arm.

10.4. Analysis of secondary outcome variables

Binary outcomes (*HIV-1 incidence, recent abuse by a sexual partner, prevalence of likely depression, inconsistent condom use, PrEP disclosure*). We will report the proportion of participants reporting these binary outcomes by randomized group and use a binomial regression model with identity link to estimate the RD and two-sided 95% CI for each outcome in the HIVST arm compared to SOC at 6 and 12 months.

Continuous outcomes (*self-efficacy, number of sexual partners*). We will report means, medians, and interquartile ranges by randomized group for these continuous outcomes, and use multivariable linear regression models, adjusting for the corresponding baseline measure (self-efficacy, number of sexual partners), to estimate effect size estimates as differences in means and two-sided 95% CIs at 6 and 12 months.

Categorical outcomes (*HIV-1 testing preference*). We will report the proportion of participants reporting the preference for clinic-based HIV-1 testing versus HIVST (combining the preferences for oral-fluid and blood-based HIVST), and also report the proportion of participants reporting the preference for oral-fluid versus blood-based (combining the preferences for clinic-based and blood-based HIVST) HIV-1 testing. We will report these proportions by randomization groups and use a binomial regression with identity link to estimate RDs and two-sided 95% CIs at 6 and 12 months.

10.5. Sub-group analyses

The following populations and sub-groups that are planned for analysis for any of the primary or secondary outcomes described above include:

- Participants in HIV-1 serodiscordant couples (N=295)

- Women (including those both in and not in HIV-1 serodiscordant couples) (N=330)
- Women not in HIV-1 serodiscordant couples (N=200)
- Age (<30 years & ≥30 years)
- Pre-COVID-19 period (pre 3/28/2020) & Post-COVID-19 period (post 3/28/2020) (exploratory)*
- Participants in HIV-1 serodiscordant couples who continue to be at risk for HIV acquisition at 12 months (exploratory)**

*We will compare data pre- and post-emergence of COVID-19 with time-varying covariates to understand if COVID-19 modifies the effect of the intervention on study outcomes. Since all participants had completed enrollment prior to the onset of COVID-19 in Kenya (3/28/2020), we will just look at the differences for the pre- and post-COVID-19 periods at 6 and 12 months. Because almost all participants had completed their 6-month follow-up visits when COVID-19 emerged in Kenya, we will likely be underpowered to measure differences between these sub-groups at 6 months.

**We will conduct this sub-group analysis only at month 12. In this sub-group analysis, we will exclude women not in HIV-1 serodiscordant couples and participants who have discontinued PrEP due to any of the following reasons (following Kenya standards for PrEP discontinuation): the participant is no longer in an HIV-1 serodiscordant partnership, the participant's HIV-positive partner has initiated and sustained ART for >6 months, or the participant's HIV-positive partner has achieved HIV viral suppression.

For the sub-groups above, the same models described above will be used at 6 and 12 months. The same non-inferiority margin of 10% will be used. Given that subgroup analyses do not have strong power, any failure to show non-inferiority in a subgroup will be considered in the context of the estimated RD and the point estimate and CI for the overall RD in the trial.

10.6. Sensitivity analysis

We will conduct a sensitivity analysis among participants in HIV-1 serodiscordant couples at 12 months to evaluate the effect of the intervention on the secondary outcomes defined above, as well as PrEP discontinuation due to a change in HIV risk following Kenya standards for PrEP discontinuation. In this analysis, we will categorize all secondary outcomes as having been achieved for participants who have self-reported discontinuing PrEP at 12 months for any of the following reasons: the participant is no longer in an HIV-1 serodiscordant partnership, the participant's HIV-positive partner has initiated and sustained ART for >6 months, or the participant's HIV-positive partner has achieved HIV viral suppression. We will report the proportion of participants reporting these binary outcomes by randomized group and will use a binomial regression model with identity link to estimate the RD and two-sided 95% CI for each outcome in the HIVST arm compared to SOC at 12 months.

Adverse Events

The total number of adverse events will be reported by arm, by grade. The proportion of participants with the following events will be compared using a Fisher's exact test:

- Physical violence, assault, or abuse
- Non-physical harassment or assault
- Unintentional or unauthorized disclosure of HIV status
- HIV self-test kit misuse (e.g., incorrectly using the HIV self-test kit)
- Suicidal thoughts or ideation
- Death

Serious adverse events (including any instance of violence, suicidality, or death) will be reported to the DSMB within 24 hours, including a report of the circumstances surrounding the adverse event. The randomization arm of the participant will be communicated from Katherine Thomas (the study statistician) to the DSMB.

All serious and non-serious adverse events will be included in interim and final monitoring reports.

11. Changes to the SAP after unblinding

A summary of changes that have been made to the SAP after unblinding is presented below. All changes are in bold.

Date: Versions	Change(s)	Reason for Change(s)
15 Dec 2021: Version 4.0 to version 4.1	**Page 14 of 18 (Section 10.4, Analysis of secondary outcome variables)** was updated to indicate that risk differences will be presented for the secondary analysis outcome variables instead of relative risks and that the effect size for each secondary outcome variable would be estimated at two timepoints: months 6 and 12. We also clarified that 95% CIs for these outcomes will be two sided**.**	We have revised the effect size estimates for secondary variables to present risk differences to be consistent with the primary outcome estimates. We have also revised the analyses to estimate separate effect sizes for months 6 and 12, as we predict that the outcomes at these timepoints are not correlated and will actually be different. Kenyan guidelines recommend that people in serodifferent partnerships discontinue PrEP after their HIV-positive partner has been on ART for at least 6 months. Therefore, we predict that many people will discontinue PrEP for this reason by month 6, and outcomes at month 12 will likely be different. Additionally, we have clarified that the 95% confidence intervals for these effect size estimates will be two-sided, as these analyses are not intended to establish noninferiority of the intervention as our primary outcome comparison does.
	**Page 7 of 18 (Section 4.2, Secondary outcomes)** was revised to update the definition of PrEP adherence at 12 months for participants in an HIV-1 serodiscordant couple. These participants will not be counted as adherent to PrEP at 12 months if they discontinue PrEP because their HIV-positive partner has initiated and sustained ART for >6 months.	We have revised the secondary outcome definition for PrEP adherence at 12 months, as we do not anticipate that the intervention will have an effect on HIV-positive partners' ART adherence; therefore, we anticipate PrEP discontinuation due to this reason will be equally distributed across study arms since the number of participants in HIV serodiscordant partnerships is the same for each study arm. Instead, we will discuss the potential limitations of our analyses when we present the findings, and we have added a subgroup analysis and a sensitivity analysis to better understand the effect of the intervention on PrEP discontinuation.
	**Page 15 of 18 (Section 10.5, Sub-group analyses)** was updated to include an additional exploratory analysis among participants in HIV-1 serodiscordant couples who continue to be at risk for HIV acquisition at 12 months.	In this subgroup analysis, we will exclude singly enrolled women and participants who discontinue PrEP at 12 months due to no longer being in an HIV-1 serodiscordant partnership or if their partner has sustained ART for >6 months or achieved viral suppression. This analysis will evaluate the true association between the intervention and PrEP refilling and adherence by removing the effects of partners' behaviors on HIV risk.
	**Page 15 of 18 (Section 10.6, Sensitivity**	This sensitivity analysis was added to

	**analysis)** was updated to include a sensitivity analysis among HIV-1 serodiscordant couples in which all secondary outcomes at 12 months among those who have discontinued PrEP following Kenya guidelines (i.e., due to no longer being in an HIV-1 serodiscordant partnership or if their partner has sustained ART for >6 months or achieved viral suppression) will be classified as having been achieved.	determine if the effects of the intervention change when discontinuing PrEP due to a reduction in HIV risk is also counted as a success.
12 Jan 2022: Version 4.1 to version 4.2	**Page 7 of 18 (Section 4.2. Secondary outcomes)** was revised to change the definition of the secondary outcome of 'persistence in refilling PrEP at 12 months' to reflect the same measure at 6 months. The definition was changed from 2 outcome measures: (1) Any PrEP refill in past 6 months and (2) Two or more PrEP refills between enrollment and 12-month visit.	We've changed the definition of this measure back to the definition of the variable from an earlier version of the SAP, which was the correct definition. We have also added an additional outcome measure to determine the persistence in PrEP refilling at both 6- and 12-month visits.
28 Mar 2022: Version 4.2 to version 4.3	**Page 14 of 18 (Section 10.4. Analysis of secondary outcome variables)** was revised to also report means for each continuous outcome and to remove the language about mixed linear regression models.	We have fixed two errors that were discovered for the analyses of continuous outcomes.
