# Supplementary material for: Effect of 6-Month HIV Preexposure Prophylaxis Dispensing With Interim Self-testing on Preexposure Prophylaxis Continuation at 12 Months: A Randomized Noninferiority Trial
Source: JAMA Netw Open. 2023 Jun 15;6(6):e2318590. doi: 10.1001/jamanetworkopen.2023.18590 (PMC10273023; doi:10.1001/jamanetworkopen.2023.18590)
Supplement: Supplement 2. — eTable. Sensitivity Analyses for All Participants and Subgroups [file jamanetwopen-e2318590-s002.pdf]

Supplemental Online Content

Ortblad KF, Bardon AR, Mogere P, et al. Effect of 6-month HIV preexposure prophylaxis dispensing with interim self-testing on preexposure prophylaxis continuation at 12 months. *JAMA Netw Open.* 2023;6(6):e2318590. doi:10.1001/jamanetworkopen.2023.18590

eTable. Sensitivity Analyses for All Participants and Subgroups

This supplemental material has been provided by the authors to give readers additional information about their work.

© 2023 Ortblad KF et al. *JAMA Network Open.*

eTable. Sensitivity Analyses for All Participants and Subgroups

Outcome	6-mo PrEP + HIVST[a] No. (%)	SOC No. (%)	6-mo PrEP + HIVST[a] vs. SOC
**All participants**			
**1. PrEP adherence threshold**	**N=329**	**N=166**	**RD (1-sided 95% CI LB)**
Returned to clinic[b]	241 (73.3)	120 (72.3)	
Adherent (≥700 fmol/punch at 12 months)	108 (32.8)	48 (28.9)	6.67% (0.30%)
Adherent (≥700 fmol/punch at 6 and 12 months)	91 (27.7)	38 (22.9)	7.53% (1.92%)
**2. HIV testing ≥2 times[a]**	**N=329**	**N=166**	**RD (1-sided 95% CI LB)**
Returned to clinic[b]	241 (73.3)	120 (72.3)	
Tested for HIV (≥2 times since enrollment)	238 (72.3)	119 (71.7)	0.24% (-6.75%)
**Subgroup 1: HIV serodifferent couples**			
**1. PrEP adherence threshold**	**N=196**	**N=99**	**RD (1-sided 95% CI LB)**
Returned to clinic[b]	145 (74.0)	78 (78.8)	
Adherent (≥700 fmol/punch at 12 months)	81 (41.3)	43 (43.4)	-2.11% (-12.14%)
Adherent (≥700 fmol/punch at 6 and 12 months)	70 (35.7)	35 (35.4)	0.36% (-9.34%)
**2. HIV testing ≥2 times[a]**	**N=196**	**N=99**	**RD (1-sided 95% CI LB)**
Returned to clinic[b]			
Tested for HIV (≥2 times since enrollment)	144 (73.5)	77 (77.8)	-4.30% (-12.90%)
**Subgroup 2: Singly enrolled women**			
**1. PrEP adherence threshold**	**N=133**	**N=67**	**RD (2-sided 95% CI)**
Returned to clinic[b]	96 (72.2)	42 (62.7)	
Adherent (≥700 fmol/punch at 12 months)	27 (20.3)	5 (7.5)	12.84% (3.55%, 22.13%)
Adherent (≥700 fmol/punch at 6 and 12 months)	21 (15.8)	3 (4.5)	11.31% (3.38%, 19.24%)
**2. HIV testing ≥2 times[a]**	**N=133**	**N=67**	**RD (2-sided 95% CI)**
Returned to clinic[b]	96 (72.2)	42 (62.7)	
Tested for HIV (≥2 times since enrollment)	94 (70.7)	42 (62.7)	7.99% (-5.94%, 21.92%)

Abbreviations: confidence interval (CI); HIV self-testing (HIVST); lower bound (LB); risk difference (RD); standard-of-care (SOC); tenofovir-diphosphate (TFV-DP)

RDs measured using binomial regression models with identity links, adjusted for study population at enrollment (e.g., men in HIV serodifferent couples, women in HIV serodifferent couples, and women singly enrolled).

© 2023 Ortblad KF et al. *JAMA Network Open.*

[a]Participants randomized to these intervention groups received 6-monthly PrEP dispensing + interim HIVST (either oral-fluid or blood-based) with semiannual clinic visits.

[b]We included all follow-up visits assigned as 12-month visits by study staff and which occurred prior to extensive follow-up procedures (extensive follow-up occurred when a participant had not returned for a 12-month visit by 15 months post-enrollment).

© 2023 Ortblad KF et al. *JAMA Network Open.*
