# Supplementary material for: Effect of 6-Month HIV Preexposure Prophylaxis Dispensing With Interim Self-testing on Preexposure Prophylaxis Continuation at 12 Months: A Randomized Noninferiority Trial
Source: JAMA Netw Open. 2023 Jun 15;6(6):e2318590. doi: 10.1001/jamanetworkopen.2023.18590 (PMC10273023; doi:10.1001/jamanetworkopen.2023.18590)
Supplement: Supplement 3. — Data Sharing Statement [file jamanetwopen-e2318590-s003.pdf]

Data Sharing Statement

Ortblad. Effect of 6-Month HIV Preexposure Prophylaxis Dispensing With Interim Self-testing on Preexposure Prophylaxis Continuation at 12 Months. *JAMA Netw Open*. Published June 15, 2023. doi:10.1001/jamanetworkopen.2023.18590

Data
Data available: Yes
Data types: Deidentified participant data
How to access data: kortblad@fredhutch.org
When available: With publication

Supporting Documents
Document types: None

Additional Information
Who can access the data: Data will be made available upon author request and under appropriate data sharing agreements who provide a methodologically sound proposal.
Types of analyses: Meta-analyses and systematic literature reviews; secondary analysis upon request and discussion with Principal Investigators
Mechanisms of data availability: with investigator support and a signed data use agreement
